# Co-expression network analysis predicts a key role of microRNAs in the adaptation of the porcine skeletal muscle to nutrient supply

**DOI:** 10.1186/s40104-019-0412-z

**Published:** 2020-01-17

**Authors:** Emilio Mármol-Sánchez, Yuliaxis Ramayo-Caldas, Raquel Quintanilla, Tainã Figueiredo Cardoso, Rayner González-Prendes, Joan Tibau, Marcel Amills

**Affiliations:** 1 Centre for Research in Agricultural Genomics (CRAG), CSIC-IRTA-UAB-UB, Universitat Autònoma de Barcelona, 08193 Bellaterra, Spain; 2 Animal Breeding and Genetics Program, Institute for Research and Technology in Food and Agriculture (IRTA), Torre Marimon, 08140 Caldes de Montbui, Spain; 3 Present address: Embrapa Pecuária Sudeste, Empresa Brasileira de Pesquisa Agropecuária (EMBRAPA), São Carlos, SP 13560-970 Brazil; 4 Department of Animal Science, Universitat de Lleida - Agrotecnio Center, 25198 Lleida, Spain; 5 Departament de Ciència Animal i dels Aliments, Universitat Autònoma de Barcelona, 08193 Bellaterra, Barcelona, Spain

**Keywords:** Co-expression analysis, lincRNAs, microRNAs, Pig, Regulatory impact factor, Skeletal muscle

## Abstract

**Background:**

The role of non-coding RNAs in the porcine muscle metabolism is poorly understood, with few studies investigating their expression patterns in response to nutrient supply. Therefore, we aimed to investigate the changes in microRNAs (miRNAs), long intergenic non-coding RNAs (lincRNAs) and mRNAs muscle expression before and after food intake.

**Results:**

We measured the miRNA, lincRNA and mRNA expression levels in the *gluteus medius* muscle of 12 gilts in a fasting condition (AL-T0) and 24 gilts fed *ad libitum* during either 5 h. (AL-T1, *N* = 12) or 7 h. (AL-T2, *N* = 12) prior to slaughter. The small RNA fraction was extracted from muscle samples retrieved from the 36 gilts and sequenced, whereas lincRNA and mRNA expression data were already available. In terms of mean and variance, the expression profiles of miRNAs and lincRNAs in the porcine muscle were quite different than those of mRNAs. Food intake induced the differential expression of 149 (AL-T0/AL-T1) and 435 (AL-T0/AL-T2) mRNAs, 6 (AL-T0/AL-T1) and 28 (AL-T0/AL-T2) miRNAs and none lincRNAs, while the number of differentially dispersed genes was much lower. Among the set of differentially expressed miRNAs, we identified ssc-miR-148a-3p, ssc-miR-22-3p and ssc-miR-1, which play key roles in the regulation of glucose and lipid metabolism. Besides, co-expression network analyses revealed several miRNAs that putatively interact with mRNAs playing key metabolic roles and that also showed differential expression before and after feeding. One case example was represented by seven miRNAs (ssc-miR-148a-3p, ssc-miR-151-3p, ssc-miR-30a-3p, ssc-miR-30e-3p, ssc-miR-421-5p, ssc-miR-493-5p and ssc-miR-503) which putatively interact with the *PDK4* mRNA, one of the master regulators of glucose utilization and fatty acid oxidation.

**Conclusions:**

As a whole, our results evidence that microRNAs are likely to play an important role in the porcine skeletal muscle metabolic adaptation to nutrient availability.

## Background

The majority of nutrigenomic studies in domestic animals have investigated the effects of dietary factors on the mean expression of messenger RNAs (mRNAs) [1], whereas the potential consequences of nutrition on the expression profiles of microRNAs (miRNAs) and long intergenic non-coding RNAs (lincRNAs) have not been explored in depth. Although changes in the expression of porcine genes in response to dietary and genetic factors have been reported in previous studies [2–6], the regulatory co-expression networks underlying such changes have not been fully elucidated yet [3, 7, 8]. Moreover, gene expression variance (GEV), also referred as gene dispersion, has been often overlooked, being considered just as experimental noise without any biological significance [9]. Few methods have been explicitly designed for modeling GEV across samples in RNA-Seq experiments [10, 11], despite the fact that changes in gene expression in response to a specific stimulus might have a biologically meaningful individual component that should not be confounded with experimental noise. Indeed, metabolic responses to nutritional factors are often driven by complex signaling pathways and gene-to-gene interactions that are not necessarily identical across the whole cohort of analyzed biological replicates, adding an intrinsic source of variation in gene expression patterns that is often ignored or modeled as a constant variable [11]. A widely accepted estimator of GEV is the biological coefficient of variation (BCV) [12]. In contrast with the canonical coefficient of variation (CV), the BCV effectively integrates both technical and biological variability, thus avoiding the dependence on count size that CV commonly shows.

When the expression patterns of two experimental groups are compared, differences in the magnitudes of average gene expression (differential gene expression) and GEV (differential gene dispersion) can be observed. Differential dispersion might be particularly useful to identify regulatory changes induced by the experimental factor under study. For instance, it is assumed that genes with low GEV are central members of signal transduction pathways while those with high GEV tend to occupy more peripheral positions in gene networks [13]. However, the central or peripheral position of a given gene in a network is not necessarily stable across time and it could also be altered by the experimental factor being analyzed. Differential dispersion could be a useful parameter to detect such source of biological variation as well as to infer its potential consequences.

In a previous study, we investigated how the patterns of mRNA expression change in response to food intake by comparing the muscle transcriptomes of fasting vs. fed gilts [5]. Herewith, we wanted to determine how the expression profiles of miRNAs and lincRNAs vary in response to nutrient supply by using mRNA profiles as a reference [5]. This analysis took into consideration both changes in the mean (differential expression) and the variance (differential dispersion) of gene expression. Moreover, we have used a co-expression network approach to elucidate potential regulatory interactions between expressed miRNAs and differentially expressed (DE) mRNA genes as well as to investigate the relationship between gene co-expression modules and meat quality and fatty acid composition traits recorded in the *gluteus medius* skeletal muscle of Duroc pigs.

## Materials and methods

### Animal material and phenotypic recording

**Fig. 1:**
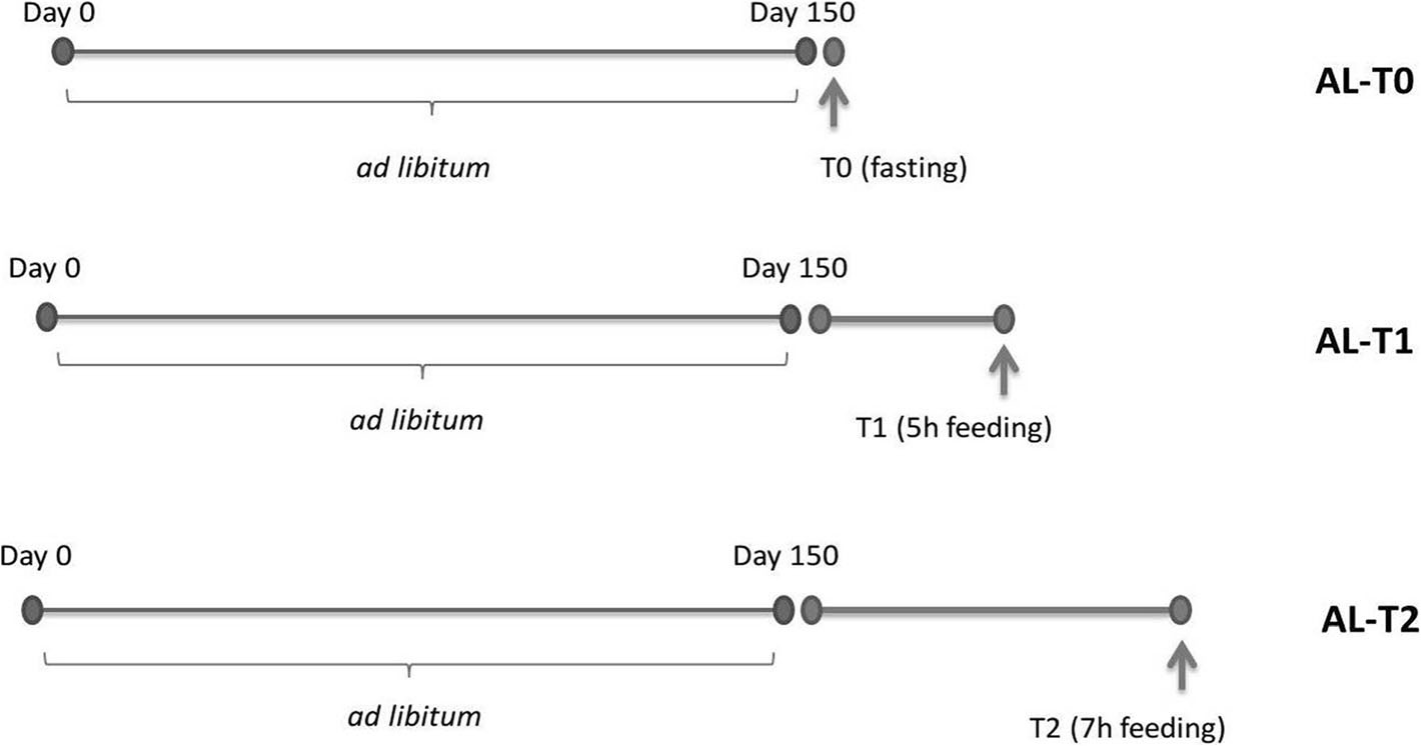
Depiction of the experimental design used in our study. Gilts were fed *ad libitum* (*N* = 36, *N* = 12 per group) with a commercial feeding diet during the whole growth period. Prior to slaughter, the 36 gilts were fasted for 12 h. The day of slaughter, 12 gilts (AL-T0) were killed under fasting conditions. The remaining 24 gilts were fed during 5 h. (AL-T1) and 7 h. (AL-T2) and they were subsequently slaughtered

The Duroc pig population used in the current work has been previously described [5]. Thirty-six female Duroc piglets were transported to the IRTA-Pig Experimental Farm at Monells (Girona, Spain) after weaning (age = 3–4 weeks). Gilts were kept in transition devices and fed *ad libitum* with a standard transition diet until they reached approximately 2 months of age (around 18 kg of live weight). Subsequently, all gilts were transferred to fattening pens, where they were housed individually and fed *ad libitum* until reaching approximately 155 d of age. Nutritional details about the feed provided to gilts between 60 and 155 d have been previously reported in [6]. During fattening (60 to 125 d), gilts received feed *ad libitum* with 14.6% crude protein, 4.25% crude fat, 4.8% crude fiber, 4.9% ashes, 0.92% lysine, 0.58% methionine + cysteine and 3190 kcal/kg. During the finishing period (126 to 155 d), gilts were also fed *ad libitum* with a diet containing 14.4% crude protein, 5.53% crude fat, 5.1% crude fiber, 4.9% ashes, 0.86% lysine, 0.53% methionine + cysteine and 3238 kcal/kg. Gilts were slaughtered in the IRTA Experimental Slaughterhouse in Monells (Girona, Spain) in accordance with relevant Spanish welfare regulations. Before slaughter, the 36 gilts were fasted for 12 h. Subsequently, 12 gilts were slaughtered in a fasting condition (AL-T0, *N* = 12), and the remaining ones were slaughtered 5 h. (AL-T1, *N* = 12) and 7 h. (AL-T2, *N* = 12) after receiving food. High concentrations of CO_2_ were used to stun the gilts before bleeding. After slaughter, samples of the *gluteus medius* skeletal muscle were taken from the 36 gilts, submerged in RNAlater (Thermo Fisher Scientific, Barcelona, Spain) and stored at − 80 °C. The whole experimental design used in the current work is depicted in Fig. 1.

Phenotypes listed in Additional file 1: Table S1 were recorded in the 36 Duroc gilts. Meat quality traits were measured as described in [14, 15]. Total muscle cholesterol content was determined following Cayuela et al. [16], whereas intramuscular fatty acids content and composition were determined in accordance with previous reports [17].

### RNA isolation, library preparation and sequencing of small RNAs

The *gluteus medius* skeletal muscle RNA-Seq data set employed in the analysis of lincRNA and mRNA expression comprised a total of 36 individuals (12 AL-T0, 12 AL-T1 and 12 AL-T2 gilts). Details about the RNA extraction and sequencing protocols can be found in [5]. Briefly, *gluteus medius* skeletal muscle samples were pulverized and subsequently homogenized in 1 mL of TRI Reagent (Thermo Fisher Scientific, Barcelona, Spain). The RiboPure kit (Ambion, Austin, TX) was used to isolate the total RNA fraction, and its concentration and purity were determined with a Nanodrop ND-1000 spectrophotometer (Thermo Fisher Scientific, Barcelona, Spain). RNA integrity was assessed with a Bioanalyzer-2100 equipment (Agilent Technologies Inc., Santa Clara, CA) by using the Agilent RNA 6000 Nano Kit (Agilent Technologies, Inc., Santa Clara, CA). Libraries were prepared with the TruSeq SBS Kit v3-HS (Illumina Inc. CA) and paired-end sequenced (2 × 75 bp) in a HiSeq 2000 platform (Illumina Inc., CA) at the Centro Nacional de Análisis Genómico (https://www.cnag.crg.eu).

In the present study, we have generated an additional *gluteus medius* skeletal muscle RNA-Seq data set specifically targeting small RNAs and comprising the same 36 individuals cited above. Total RNA was purified as reported above. The percentage of small-RNA over total RNA was determined with the Agilent Small RNA Kit (Agilent Technologies Inc., Santa Clara, CA). All 36 samples met the quality threshold (i.e. 0.2–2 μg total RNA with RIN > 7 and miRNA percentage over total RNA > 0.5%) to be sequenced in Sistemas Genómicos S.L. (https://www.sistemasgenomicos.com). Individual libraries for each sample (*N* = 36) were prepared with the TruSeq Small RNA Sample Preparation Kit (Illumina Inc., CA) according to the protocols of the manufacturer. Small RNA libraries were then subjected to single-end (1 × 50 bp) sequencing in a HiSeq 2500 platform (Illumina Inc., CA).

### Quality assessment, mapping and count estimation

Quality control of paired-end reads was performed with the FASTQC software (Babraham Bioinformatics, http://www.bioinformatics.babraham.ac.uk./projects/fastqc/) and filtered reads were trimmed for any remaining sequencing adapters with the Trimmomatic v. 0.22 tool [18], as described in [5, 6]. In the case of single-end sequenced reads derived from small RNA molecules, sequencing adapters were trimmed and filtered with the Cutadapt software [19], and reads outside a window of 15–25 nucleotides were discarded. Paired-end trimmed raw reads from RNA-Seq sequences were mapped to the porcine Sscrofa.11.1 reference assembly by using the HISAT2 aligner [20] with default parameters. The Stringtie software [21] was subsequently employed to estimate mRNA and lincRNA abundances. Single-end trimmed raw reads derived from small RNAs were also mapped to the Ssscrofa.11.1 assembly with the Bowtie Alignment v.1.2.1.1 software [22], and the following specifications for aligning short miRNA reads were taken into consideration: 1) allowing no mismatches in the alignment, 2) removing reads with more than 20 putative mapping sites and 3) reporting first single best stratum alignment (*bowtie -n 0 -l 25 -m 20 -k 1 --best --strata*). The featureCounts software tool [23] was then used to summarize counts of unambiguously mapped reads from miRNA-Seq sequences.

### Differential expression and differential dispersion estimates

Raw expression matrices generated on the basis of count estimates obtained with Stringtie (mRNAs and lincRNAs) or featureCounts (miRNAs) [21, 23] were normalized with the trimmed mean of M-values normalization method [24]. Sequencing depth and read count per gene were calculated for each sequenced sample (Additional file 15: Figure S1). On the basis of this analysis, the AL-T0 7197 sample was removed from RNA-Seq and miRNA-Seq count matrices due to the low read coverage observed in the RNA-Seq sequencing data set. The presence of influential outliers for each estimate of gene expression was corrected by capping expression values laying outside the boundaries of 1.5 times inter-quartile range per gene and fitting them within the 10^th^ and 90^th^ percentiles. For estimating GEV, the BCV was computed for each detected annotated gene as described in the *edgeR* protocol [25], and further discussed in [12]. The BCV encapsulates all sources of inter-library variation between replicates, including the contribution of library preparation biases [12].

Differentially expressed (DE) and dispersed (DD) genes were determined by comparing the means and variances of gene expression in the two AL-T0/AL-T1 and AL-T0/AL-T2 contrasts. Only mRNAs and miRNAs showing an average expression value above 1 count-per-million (CPM) in at least 50% (*N* = 12) of the samples (considering all AL-T0, AL-T1 and AL-T2 samples) were retained for further analyses. Because lincRNAs are much less expressed than mRNAs and miRNAs, all lincRNAs (*N* = 352) annotated in the Sscrofa11.1 reference assembly (v. 97) were considered for differential expression and dispersion analyses (a filtering step imposing an expression threshold above 1 CPM would have implied the removal of as much as 80% of annotated lincRNA loci). The *edgeR* [25] and *MDSeq* [10] packages with default parameters were used for performing differential expression and dispersion analyses, respectively. The *edgeR* protocol uses the quantile-adjusted conditional maximum likelihood method for detecting differences in gene expression between two groups. Once negative binomial models are fitted to the input counts and dispersion estimates are obtained, differential expression is determined by using an exact test of significance. Correction for multiple hypothesis testing is implemented by using the Benjamini-Hochberg false discovery rate approach [26]. The *MDSeq* method implements a re-parametrization of the real-valued negative binomial distribution to allow the modelling of gene expression variability [10]. Correction for multiple hypothesis testing across genes is implemented with the Benjamini-Yekutieli procedure [27]. The DE and DD genes obtained with *MDSeq* and *edgeR* were considered to be significant at a fold change > |1.5| and *q*-value < 0.05.

### Gene Ontology and pathway enrichment analysis

The lists of mRNA genes detected as DE in the AL-T0/AL-T1 and AL-T0/AL-T2 contrasts were used as inputs for Gene Ontology (GO) and pathway enrichment analyses. The ClueGO v2.5.0 plug-in application [28] embedded in the Cytoscape 3.5.1 software [29] was used for determining enriched Reactome and KEGG pathways, as well as Biological Process enriched GO terms. A two-sided hypergeometric test of significance was applied for determining enriched terms and multiple testing correction for pathway enrichment analyses was implemented with a false discovery rate approach [26], whereas a Bonferroni-based multiple testing correction was used in the GO enrichment analysis.

### Building of co-expression networks

Significant connections between predicted interacting gene pairs were identified with the Partial Correlation with Information Theory (PCIT) network inference algorithm [30]. By using first-order partial correlation coefficients estimated for each trio of genes along with an information theory approach, this tool identifies meaningful gene-to-gene putative interactions. The PCIT approach has been widely used to reconstruct co-expression regulatory networks from expression data with good performance [31]. The main aim of this analysis was to determine truly informative correlations between node pairs (genes in our context), once the influence of other nodes in the network has been considered.

Pearson pairwise correlation coefficients (*r*) were calculated for each expressed miRNA and DE mRNAs in each of the two contrasts (AL-T0/AL-T1 and AL-T0/AL-T2). The *pcit* function from the PCIT R package [30, 32] was then used for detecting meaningful co-expressed gene pairs. To further identify the putative miRNA-to-mRNA interaction pairs with biological interest, a repressor effect of miRNAs on mRNA expression was assumed [33] and, in consequence, only miRNA-to-mRNA co-expressed pairs showing *r* < − 0.5 were retained. Furthermore, we only considered miRNA-to-mRNA interactions with perfect 7mer-m8 pairing between the miRNA-seed and the 3′-UTR of the putative mRNA targets, hence removing spurious miRNA-to-mRNA significant correlations with no robust biological meaning. To this end, we downloaded the full set of annotated 3′-UTR sequences in the porcine Sscrofa11.1 assembly available at BioMart Ensembl repositories (http://www.ensembl.org/biomart/martview/). Seed portions (2^nd^ to 8^th^ 5′ nucleotides in the mature miRNA) of the annotated set of porcine miRNAs were reverse-complemented and interrogated along the 3′-UTR sequence regions of mRNA genes by making use of the SeqKit toolkit [34]. Additionally, we selected four highly expressed and DE miRNAs (ssc-miR-148a-3p, ssc-miR-1, ssc-miR-493-5p and ssc-let-7/ssc-miR-98) and used the TargetScan webserver to evaluate the evolutionary conservation of their binding sites in the 3’UTR of predicted mRNA targets [35]. Only conserved target mRNAs with TargetScan context++ scores above the 75% percentile were considered as confidently cross-validated. The context++ score described by Agarwal et al. [35] incorporates the information of 14 estimated features in order to rank the probability of all the predicted target sites to be biologically functional.

For those mRNAs predicted to interact with miRNAs, we also investigated if they also interact with other mRNA-encoding genes. In order to focus on relevant putative mRNA-to-mRNA gene interactions, we only retained those meaningful mRNA co-expressed pairs showing |*r*| > 0.7, as assessed with the PCIT algorithm. We applied this threshold, which is more stringent than the one used for miRNA-to-mRNA interactions, because correlations between expressed mRNAs tend to be higher than those between mRNAs and miRNAs [36]. Hub genes within selected mRNA-to-mRNA gene interactions (i.e. those mRNAs showing a higher degree of meaningful connectivity according to the PCIT algorithm), were also identified by calculating a hub score per gene (*K*_*i*_), defined as:

$$ {K}_i=\frac{x_i}{\overline{X}} $$

Where *x*_*i*_ is the number of selected significant connections (|*r*| > 0.7) reported by the PCIT algorithm and *X* is the average connectivity within the mRNA-to-mRNA co-expression network among DE mRNA genes. Gene co-expression networks were visualized with the Cytoscape 3.5.1 software [29].

Besides, for each selected miRNA-to-mRNA predicted interactions, we calculated the Regulatory Impact Factor (RIF) of the corresponding miRNAs [37]. The RIF algorithm aims to identify regulator genes contributing to the observed differential expression in the analyzed contrasts. Its implementation results in two different and inter-connected RIF scores: while RIF1 score represents those transcriptional regulators that are most differentially co-expressed with the most highly abundant and highly DE genes, the RIF2 score highlights those regulators that show the most altered ability to act as predictors of the changes in the expression levels of DE genes [37]. Both RIF values capture different regulatory impact features and hence, they can be considered as two independent measurements of the putative relevance of miRNAs as gene expression regulators. The RIF1 values for each *i*^th^ regulatory factor were calculated as follows:

$$ RIF{1}_i=\frac{1}{n_{de}}\sum \limits_{j=1}^{j={n}_{de}}{PIF}_j\times {DW}_{ij}^2 $$

Where *n*_de_ is the number of DE genes and Phenotype Impact Factor (PIF) and differential wiring (DW) are denoted by:

$$ {\displaystyle \begin{array}{c} PI{F}_j=\frac{1}{2}\left(e{1}_j^2-e{2}_j^2\right)\\ {}D{W}_{ij}=r{1}_{ij}-r{2}_{ij}\end{array}} $$

being *e*1_*j*_ and *e*2_*j*_ the expression of the *j*^th^ differentially expressed gene in both conditions 1 and 2, respectively, whereas *r*1_*ij*_ and *r*2_*ij*_ represent the co-expression correlation between the *i*^th^ regulatory factor (miRNAs in our case) and the *j*^th^ DE mRNA gene in conditions 1 and 2, respectively.

The RIF2 values for each *i*^th^ regulatory factors were defined as:

$$ RIF{2}_i=\frac{1}{n_{de}}\sum \limits_{j=1}^{j={n}_{de}}\left[{\left(e{1}_j\times r{1}_{ij}\right)}^2-{\left(e{2}_j\times r{2}_{ij}\right)}^2\right] $$

The positive or negative sign of the RIF1 score is mainly determined by the magnitude of the PIF estimates, and hence is dependent on the directionality of the defined contrast (i.e. the AL-T0/AL-T2 vs. AL-T2/AL-T0 contrasts would generate RIF1 scores with opposite signs). In contrast, the sign of the RIF2 score reflects the altered ability of the regulators to act as predictors of the abundance of DE genes [37].

### Association between muscle phenotypes and weighted gene co-expression networks

Significant associations between key co-expressed genes and meat quality and fatty acids composition traits measured in the *gluteus medius* skeletal muscle samples (Additional file 1: Table S1) were determined with the weighted gene correlation network analysis (WGCNA) approach [38]. We used the WGCNA R package [38] for building signed weighted gene co-expression modules based on mRNA and miRNA genes present in the AL-T0/AL-T1 and AL-T0/AL-T2 count matrices and displaying a minimum expression of 1 CPM in at least 50% of samples. Weighted adjacency matrices were built for each expression data set by using a power soft threshold (β) = 16, as recommended by Langfelder and Horvath [38] for estimating signed correlations based on the number of replicates used in our experimental design. The obtained weighted adjacency matrices were subsequently transformed into topological overlapping matrices (TOM) and corresponding dissimilarities were calculated to minimize the effect of noise and spurious co-expression patterns. Hierarchical clustering was then applied to the dissimilarity matrices (1-TOM) and co-expressed genes were merged into modules through dynamic tree branch cutting. Highly inter-connected modules were finally merged by calculating their eigengenes and setting a minimum height cut of 0.25 and a minimum module size of 30 genes for each identified gene co-expression module.

To further elucidate whether the inferred gene co-expression modules were significantly associated with the variation of meat quality and fatty acids composition traits (Additional file 1: Table S1), module eigengenes (MEs) were defined as the first principal component calculated with the Principal Component Analysis (PCA) algorithm. In this way MEs summarize the co-expression patterns of all genes within each module into a single variable. Measured phenotypes were then correlated with each defined ME. Correlated phenotype-module pairs were considered to be significant when *P*-value < 0.05. Co-expressed miRNA-only modules were discarded for further analyses. A Student asymptotic *P*-value approach was finally used for determining the significance of the contribution of each gene within the co-expression modules to the correlation coefficient between MEs and each one of the recorded phenotypes. Relevant genes within significant modules were selected based on the estimates of gene significance (GS, *P*-value < 0.05) obtained for each phenotype-module significant association.

Additionally, hub genes within each detected gene co-expression module showing significant correlations with phenotypic traits were assessed. WGCNA inferred networks were converted to edge graphs by using the RNAseqDE wrapper R package (https://github.com/jtlovell/RNAseqDE). Subsequently, hub scores for each gene in the selected co-expression modules were calculated by computing the scaled Kleinberg’s hub centrality score as described in the igraph tool (https://igraph.org) [39].

## Results

### Comparing the expression patterns of coding and non-coding RNAs expressed in the porcine skeletal muscle

The RNA-Seq data set employed for mRNA and lincRNA quantification encompassed an average of 48.6 million paired-end reads per sample, and approximately 93% of them mapped successfully to the Sscrofa11.1 assembly. Roughly, 76% of unambiguously mapped reads were assigned to annotated features (genes) after quantification. With regard to the miRNA-Seq experiment, an average of 8.2 million single-end reads per sample were generated, which were reduced to approximately 6.8 million reads per sample after quality-check and adapter trimming. From these, approximately 77% mapped to the porcine assembly, and an average of 42% single-end mapped reads were successfully assigned to annotated microRNAs in the Sscrofa11.1 assembly. The accuracy of the RNA-Seq procedures employed in the current work were previously validated by Cardoso et al. [40], analyzing the differential expression of eight genes based on RNA-Seq results and real-time quantitative PCR measurements of gene expression. Such comparison showed a high concordance between the results obtained with these two independent methods [40].

**Fig. 2:**
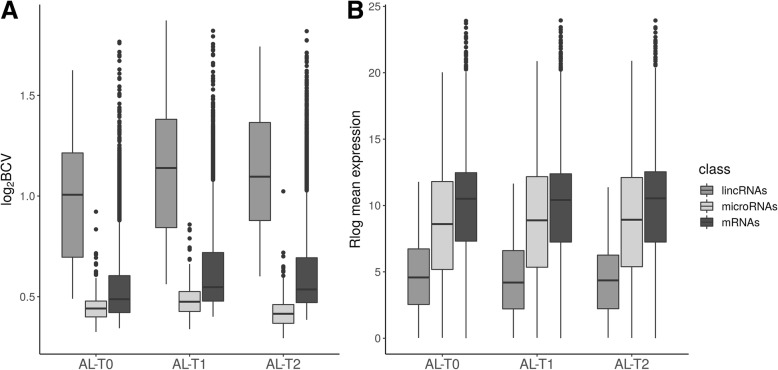
Expression variability and quantification of expression levels of mRNAs, microRNAs and lincRNAs. **a** Biological Coefficient of Variation (BCV) distribution across transcript types within each analyzed group. **b** *DESeq2* regularized log_2_ mean expression (rlog) values across transcript types within each analyzed group

**Fig. 3:**
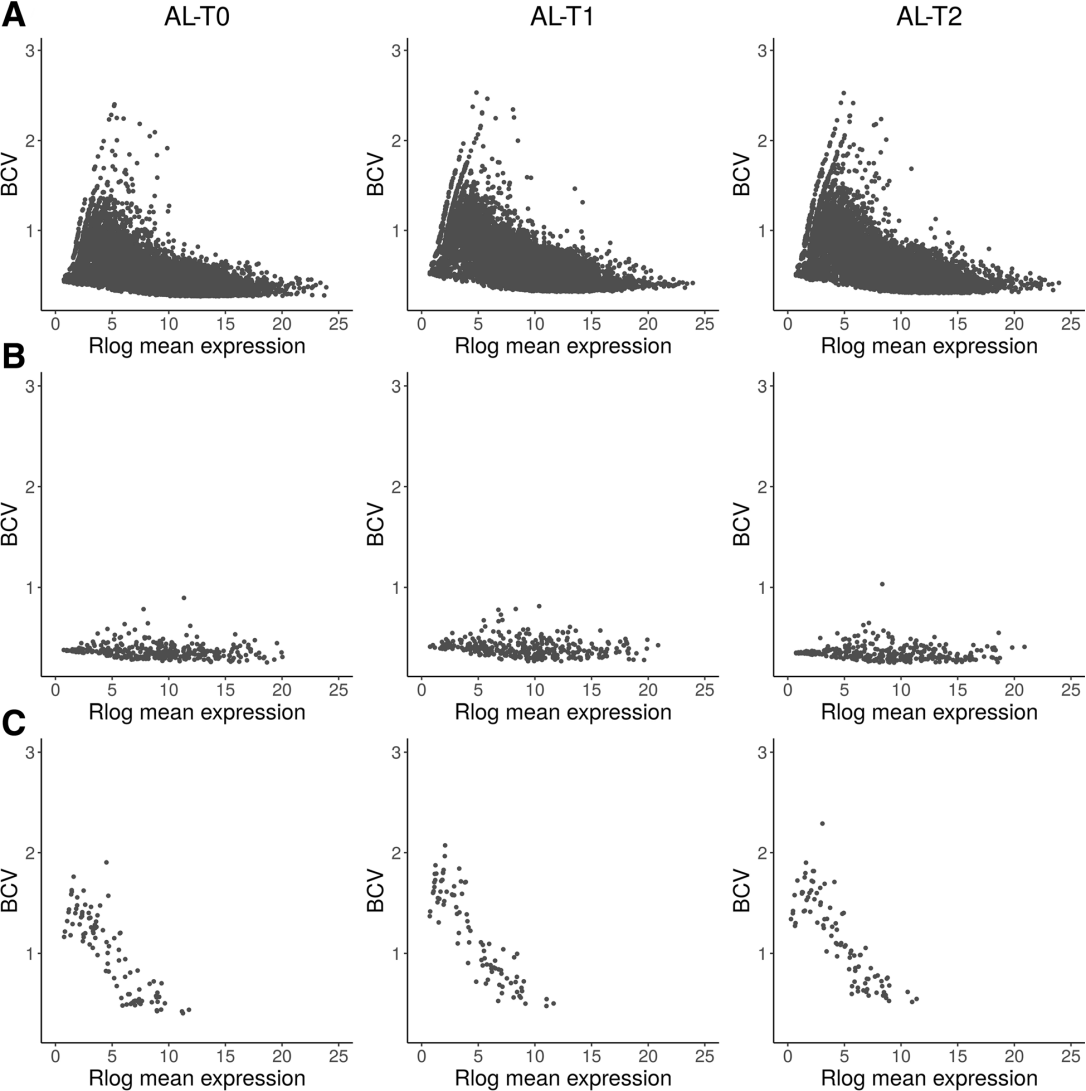
Biological Coefficient of Variation (BCV) vs. *DESeq2* regularized log_2_ mean expression (Rlog) of (**a**) mRNAs, (**b**) microRNAs and (**c**) lincRNAs in each of the analyzed groups (AL-T0, AL-T1 and AL-T2)

**Fig. 4:**
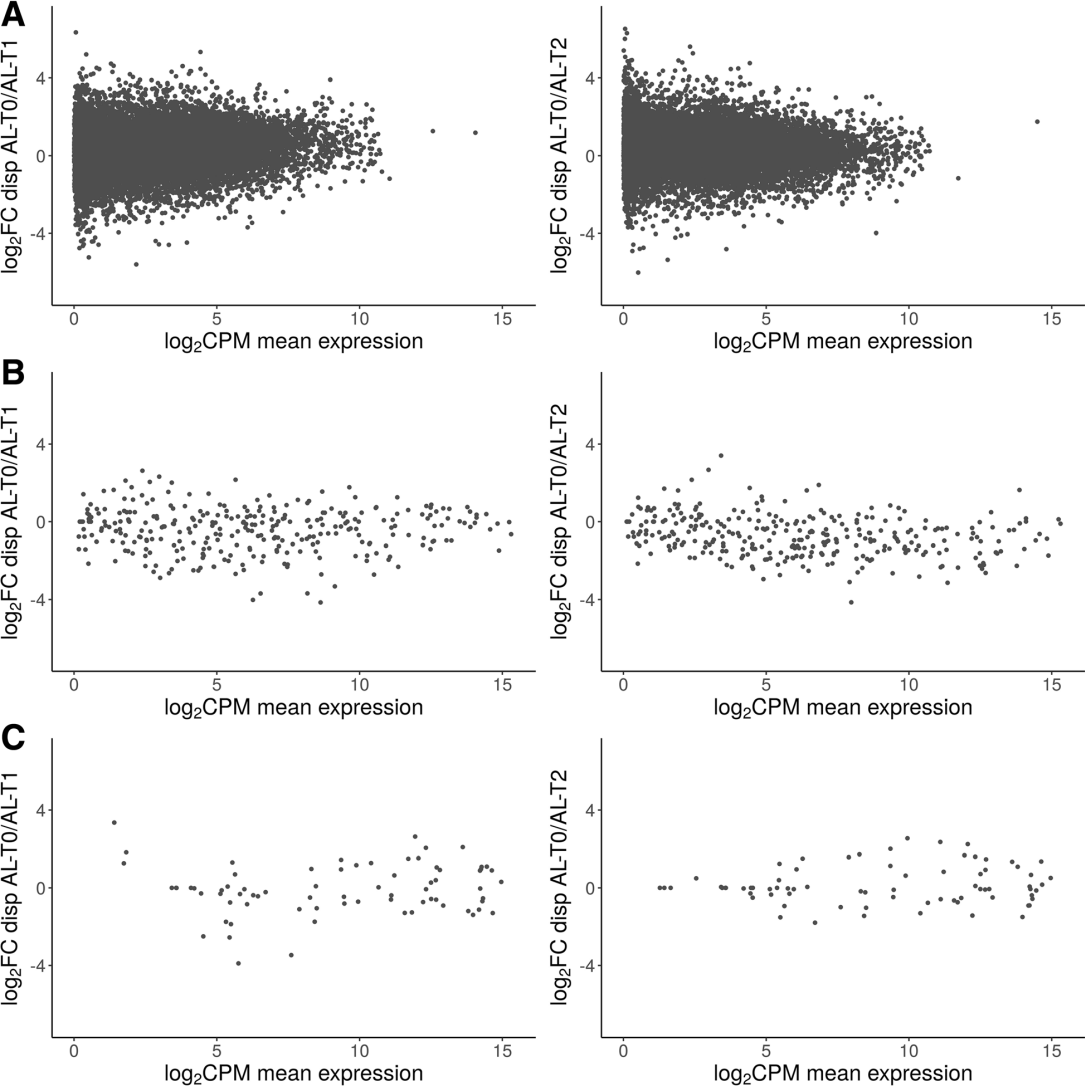
Log_2_ Fold change (FC) of the dispersion values estimated with *MDSeq* tools vs. log_2_ mean expression (counts-per-million, CPM) of (**a**) mRNAs, (**b**) microRNAs and (**c**) lincRNAs expression patterns in the AL-T0/AL-T1 (left column) and AL-T0/AL-T2 contrasts (right column)

We have characterized and compared the muscle expression profiles of lincRNAs, miRNAs and mRNAs in three groups of pigs (Fig. 1): AL-T0 (fasted), AL-T1 (5 h after feeding) and AL-T2 (7 h after feeding). The computed BCVs measuring the range of variability in gene expression across biological replicates within the same group were markedly elevated for lincRNAs, moderate for mRNAs and low for miRNAs, which ultimately showed a very stable and homogeneous expression profile across samples (Fig. 2a). Moreover, as expressed by the regularized log_2_ (Rlog) transformation of gene counts according to Love et al. [41], the average expression of lincRNAs was much lower than that of mRNAs, while miRNAs occupied an intermediate position between these two extremes (Fig. 2b). In general, lowly expressed genes displayed higher BCVs than genes with high levels of expression (Fig. 3). This pattern was especially relevant for mRNAs (Fig. 3a), with an average estimated background BCV of 0.53 (i.e. 53% of mean variability in gene expression across biological replicates expected for mRNA genes), and lincRNAs (mean BCV = 115%, Fig. 3c). In strong contrast, miRNAs showed a narrow range of gene expression variability (mean BCV = 37%). Indeed, we did not detect miRNA genes with extremely high BCV values even when we considered miRNAs expressed at marginal levels below 1 CPM (Fig. 3b). With *MDSeq* tool [10], we computed fold-changes (FC) for dispersion estimates. For each contrast, log_2_FC dispersion values were then plotted against log_2_CPM gene expression values (Fig. 4). In general, protein-coding genes with medium to low expression levels (Fig. 4a) showed higher dispersion FC values than those that were highly expressed. This antagonistic relationship was much less obvious for miRNAs or lincRNAs than for mRNAs (Fig. 4b, c).

### Identification of differentially expressed and dispersed genes

**Fig. 5:**
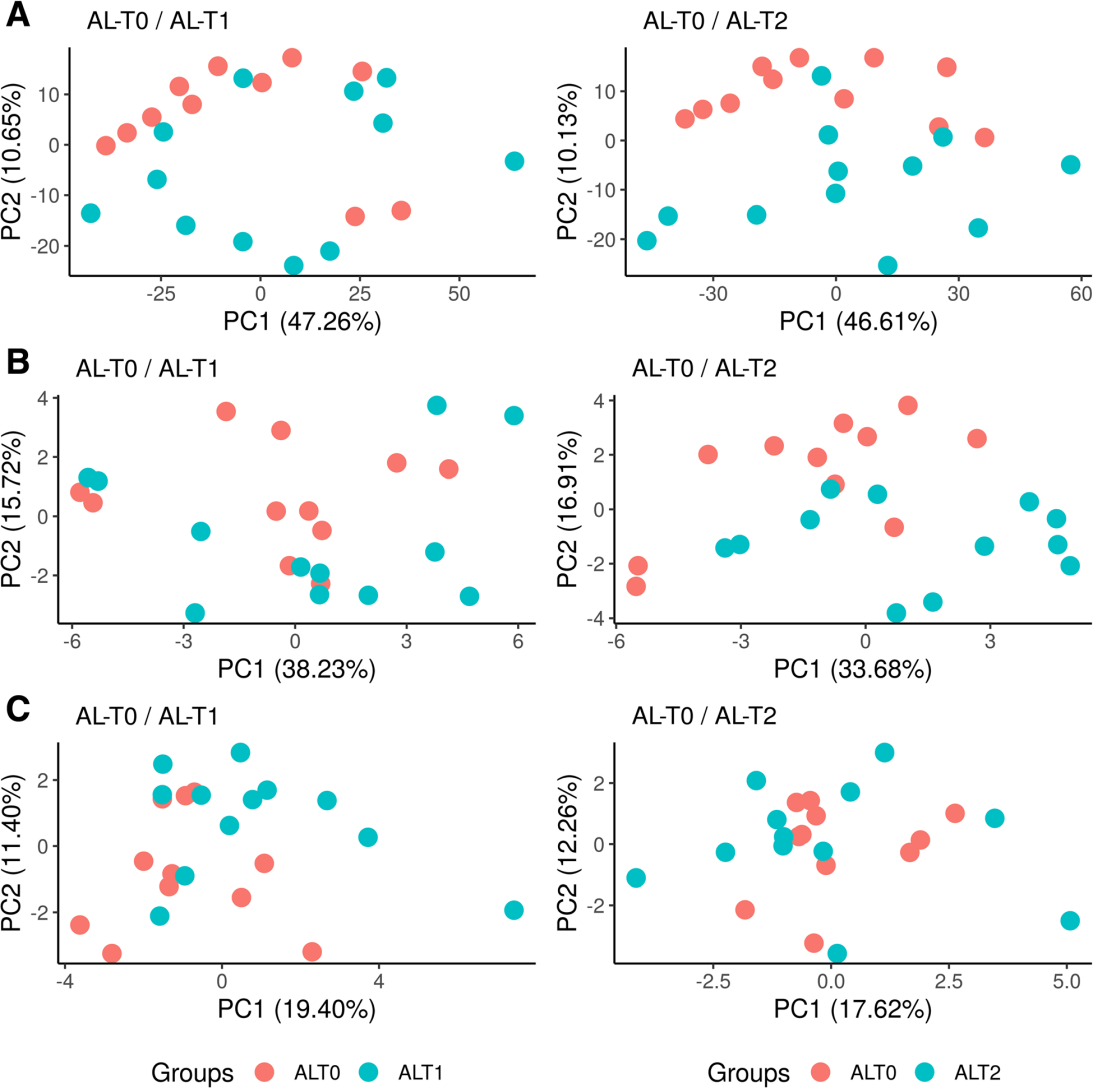
Principal Component Analysis (PCA) clustering of *gluteus medius* skeletal muscle samples (11 AL-T0, 12 AL-T1 and 12 AL-T2 gilts) according to the expression profiles of (**a**) mRNAs, (**b**) microRNAs and (**c**) lincRNAs

Principal component analysis showed a clear clustering of samples according to their group of origin (AL-T0, AL-T1 and AL-T2) when we considered mRNA expression patterns (Fig. 5a), and this was particularly true in the AL-T0/AL-T2 contrast. This outcome agrees well with previously reported results using the same experimental data [5]. In contrast, the clustering of samples based on their miRNA expression patterns was more diffuse (Fig. 5b), and in the case of lincRNAs, no evident pattern of clustering was observed (Fig. 5c). This lack of sample clustering could be due, at least in part, to the low and very low numbers of annotated pig miRNAs and lincRNAs, respectively. Moreover, the highly variable expression of lincRNAs across samples could also contribute to this lack of clustering. Joint PCA clustering considering all three contrast groups is depicted in Additional file 15: Figure S2.

As previously said, statistical analyses for DE and DD miRNA and mRNA genes were restricted to loci with expression levels above 1 CPM in each contrast and in at least 50% (*N* = 12) of the samples (each contrast includes 23 samples), whereas all annotated lincRNAs, irrespective of their expression levels, were considered. These filtering criteria reduced approximately by half the number of analyzed mRNAs, i.e. 10,648 (AL-T0/AL-T1) and 10,714 (AL-T0/AL-T2,) expressed mRNAs from a total of 22,342 annotated protein-coding genes were selected for further analyses. Regarding miRNAs, 35% of annotated miRNAs did not reach the expression threshold of 1 CPM (286 expressed miRNAs out of 442 annotated miRNA genes in both AL-T0/AL-T1 and AL-T0/AL-T2).

Differential expression and/or dispersion results generated with *MDSeq* and *edgeR* approaches reflected evident changes in the skeletal muscle transcriptomic profile of pigs after feed intake. These changes were particularly intense in the case of mRNA genes, with 149 and 435 DE mRNAs in AL-T0/AL-T1 and AL-T0/AL-T2, respectively (Additional file 2: Table S2). Moreover, 6 and 28 miRNAs (*q*-value < 0.05; |FC| > 1.5) were classified by *edgeR* as DE in AL-T0/AL-T1 and AL-T0/AL-T2 respectively (Table 1), whereas no lincRNAs showed significant DE in any of the two contrasts. When we considered a less stringent FC threshold for miRNAs and lincRNAs (|FC| > 1.2), we were able to recover 5 additional DE miRNAs in the AL-T0/AL-T2 contrast (Table 1). With regard to differential dispersion, 27 and 30 DD mRNAs were detected with *MDSeq* in the AL-T0/AL-T1 and AL-T0/AL-T2 contrasts, respectively (Additional file 3: Table S3), and several of these mRNAs were also differentially expressed (Additional file 2: Table S2). Few DD miRNAs (i.e. 5 in AL-T0/AL-T1 and 1 in AL-T0/AL-T2) and only two DD lincRNAs (in AL-T0/AL-T1) were detected (Table 2).

**Table 1:** microRNAs detected by *edgeR* as differentially expressed when comparing AL-T0 (fasted) gilts with their AL-T1 (5 h after eating) and AL-T2 (7 h after eating) counterparts

Contrast	miRNA	log_2_FC^b^	*P*-value	*q*-value^c^	log_2_CPM AL-T0^d^	log_2_CPM AL-T1^d^	log_2_CPM AL-T2^d^
AL-T0/AL-T1^a^	ssc-miR-7-5p	0.9978	7.56E-05	2.16E-02	6.8416	7.5115	–
	ssc-miR-374a-3p	0.8568	4.73E-04	3.81E-02	6.9201	7.5034	–
	ssc-miR-7	0.9229	5.26E-04	3.81E-02	6.4206	7.0178	–
	ssc-miR-148a-3p	0.8989	5.97E-04	3.81E-02	13.8692	14.5105	–
	ssc-miR-1	0.7686	6.66E-04	3.81E-02	16.8124	17.3183	–
	ssc-miR-32	1.2420	9.92E-04	4.73E-02	2.9824	3.6098	–
AL-T0/AL-T2^a^	ssc-miR-1285	−2.9830	3.47E-09	9.92E-07	7.9799	–	5.5849
	ssc-miR-148a-3p	1.3831	2.39E-06	2.83E-04	13.8692	–	14.9315
	ssc-miR-7-5p	1.1592	2.97E-06	2.83E-04	6.8416	–	7.6948
	ssc-miR-493-5p	0.7464	3.84E-05	2.37E-03	6.4846	–	7.1191
	ssc-miR-7	1.0724	4.14E-05	2.37E-03	6.4206	–	7.1910
	ssc-miR-22-3p	−0.9814	1.01E-04	4.20E-03	12.6857	–	11.7583
	ssc-miR-421-5p	1.2893	1.03E-04	4.20E-03	2.6775	–	3.7359
	ssc-miR-758	−0.7536	1.24E-04	4.43E-03	5.4106	–	4.5480
	ssc-miR-339	−0.8760	1.68E-04	5.34E-03	2.8919	–	2.0274
	ssc-let-7f-1	0.7735	2.36E-04	6.43E-03	14.0981	–	14.7031
	ssc-let-7f-5p	0.7641	2.74E-04	6.43E-03	12.4788	–	13.0761
	ssc-miR-374a-3p	0.9025	2.75E-04	6.43E-03	6.9201	–	7.5867
	ssc-miR-30a-3p	0.6600	3.36E-04	6.43E-03	9.8397	–	10.3833
	ssc-miR-151-3p	0.6940	3.37E-04	6.43E-03	12.3832	–	12.9732
	ssc-miR-129a-3p	−1.3858	4.09E-04	7.05E-03	4.7123	–	3.0830
	ssc-miR-296-5p	−0.9342	4.79E-04	7.61E-03	5.1094	–	3.9239
	ssc-miR-30e-3p	0.6430	7.45E-04	1.12E-02	10.8497	–	11.3840
	ssc-miR-98	0.7127	1.24E-03	1.69E-02	9.6818	–	10.2075
	ssc-let-7a-1	0.4660	1.13E-03	2.53E-02	13.7761	–	14.1002
	ssc-let-7a-2	0.4590	1.43E-03	2.53E-02	12.4875	–	12.8046
	ssc-miR-503	0.4912	1.12E-03	2.53E-02	7.8380	–	8.1776
	ssc-miR-181c	−0.6665	2.02E-03	2.56E-02	3.0770	–	2.3722
	ssc-miR-32	1.1189	2.11E-03	2.56E-02	2.9824	–	3.5525
	ssc-miR-1	0.6586	2.15E-03	2.56E-02	16.8124	–	17.2479
	ssc-miR-450b-3p	0.9689	2.78E-03	2.95E-02	1.3512	–	2.0993
	ssc-miR-136-5p	0.9319	2.78E-03	2.95E-02	3.4211	–	3.8968
	ssc-miR-7857-3p	−1.1003	3.03E-03	3.09E-02	2.3255	–	1.5283
	ssc-miR-125b	−0.5858	1.88E-03	3.20E-02	13.0432	–	12.3566
	ssc-miR-361-5p	−0.5109	3.02E-03	4.45E-02	7.4597	–	6.8061
	ssc-miR-362	−0.5567	3.45E-03	4.61E-02	6.5327	–	5.8194
	ssc-miR-218b	0.7746	4.75E-03	4.62E-02	4.8429	–	5.2796
	ssc-miR-532-3p	−0.6865	4.84E-03	4.62E-02	7.4422	–	6.5930
	ssc-miR-365-3p	−0.7367	5.36E-03	4.79E-02	9.9681	–	8.9921

^a^AL-T0: Duroc gilts in a fasting condition (*N* = 11); AL-T1: Duroc gilts slaughtered after 5 h of food intake (*N* = 12); AL-T2: Duroc gilts slaughtered after 7 h of food intake (*N* = 12)

^b^Log_2_FC: estimated log_2_ fold change mean expression levels

^c^*q*-value: *P*-value corrected for multiple testing with the Benjamini-Hochberg procedure

^d^Log_2_CPM: estimated log_2_ counts-per-million (CPM) mean expression levels in AL-T0, AL-T1 and AL-T2 groups

**Table 2:** microRNAs and lincRNAs detected by *MDSeq* as differentially expressed when comparing AL-T0 (fasted) gilts with their AL-T1 (5 h after eating) and AL-T2 (7 h after eating) counterparts

Contrast	log_2_FC^b^	*P*-value	*q*-value^c^	Log_2_CPM AL-T0^d^	Log_2_CPM AL-T1^d^	Log_2_CPM AL-T2^d^
AL-T0/AL-T1^a^						
miRNA						
ssc-miR-17-5p	−4.0190	3.20E-06	2.81E-03	6.2654	5.7652	–
ssc-miR-186-5p	−4.1486	1.66E-06	2.81E-03	8.6343	8.2410	–
ssc-miR-362	−3.6875	1.64E-05	9.48E-03	6.5327	5.8885	–
ssc-miR-451	−3.6825	2.16E-05	9.48E-03	8.1730	8.1217	–
ssc-miR-29a-3p	−3.3204	1.16E-04	4.07E-02	9.1335	8.7859	–
lincRNA						
ENSSSCG00000032301	3.3076	3.60E-05	1.32E-02	1.4722	5.8072	–
ENSSSCG00000031192	−3.8178	1.59E-04	2.93E-02	5.8864	1.7548	–
AL-T0/AL-T2^a^						
miRNA						
ssc-miR-1285	−4.1428	4.60E-06	8.04E-03	7.9799	–	5.5849

^a^AL-T0: Duroc gilts in a fasting condition (*N* = 11); AL-T1: Duroc gilts slaughtered after 5 h of food intake (*N* = 12); AL-T2: Duroc gilts slaughtered after 7 h of food intake (*N* = 12)

^b^Log_2_FC: estimated log_2_ fold change mean dispersion levels

^c^*q*-value: *P*-value corrected with the Benjamini-Yekutieli procedure

^d^Log_2_CPM: estimated log_2_ counts-per-million (CPM) mean expression levels in AL-T0, AL-T1 and AL-T2 groups

### Functional annotation and pathway enrichment of differentially expressed genes

A total of 26 Reactome and 8 KEGG significantly enriched pathways were detected in the AL-T0/AL-T1 contrast, whereas 16 Reactome and 14 KEGG enriched pathways were identified for the AL-T0/AL-T2 contrast (*q*-value < 0.05). Gene ontology biological process enrichment analyses resulted in 65 and 107 significant GO terms for AL-T0/AL-T1 and AL-T0/AL-T2, respectively. A complete list of enriched pathways and GO terms is shown in Additional files 4: Table S4 (AL-T0/AL-T1) and 5: Table S5 (AL-T0/AL-T2). Among the most highly enriched pathways, those related with circadian clock regulation appeared in both contrasts, as well as other pathways associated with myogenesis, nuclear receptor transcription or NOTCH1, and interleukin 4 and 13 signaling. Regarding the GO enriched terms, many biological processes triggered by nutrient availability after food intake were activated, such as skeletal muscle differentiation (GO:0035914), carbohydrate biosynthetic process (GO:0016051), regulation of gluconeogenesis (GO:0035947), glycogen biosynthetic process (GO:0005978), gluconeogenesis (GO:0006094), energy reserve metabolic process (GO:0006112), activation of transcription from RNA polymerase II promoter (GO:0006366), response to lipids (GO:0033993), adipose tissue development (GO:006012), regulation of fat cell differentiation (GO:0045598), circadian regulation of gene expression (GO:0032922), cellular response to external stimulus (GO:0071496), response to starvation (GO:0042594) or regulation of energy homeostasis (GO:2000505), to mention a few (Additional files 4 and 5: Table S4 and S5).

### Construction of co-expression networks and measurement of regulatory impact factors

We also aimed to determine whether the expression of miRNAs is associated with that of mRNAs in each one of the experimental contrasts. With the PCIT algorithm, we detected 24 (AL-T0/AL-T1) and 55 (AL-T0/AL-T2) miRNAs co-expressed (*r* < − 0.50) with sets of differentially expressed putative mRNA targets (Additional file 6: Table S6). For mRNA-to-mRNA connections, only meaningful co-expression relationships with |*r*| > 0.7 were considered (Additional file 7: Table S7). Hub genes showing a high degree of connectivity were prioritized by means of their estimated hub score values (*K*). A list of selected mRNA genes and their K values is available in Additional file 8: Table S8. Among the genes with the top (5%) hub scores, it is worth mentioning the following ones: (1) AL-T0/AL-T1: Rev-Erb-β (*NR1D2*), BTB domain and CNC homolog 1 (*BACH1*), ETS proto-oncogene 1 (*ETS1*) and the cAMP responsive element binding protein 1 (*CREB1*), and (2) AL-T0/AL-T2: secretory carrier membrane protein 2 (*SCAMP2*), neuraminidase 3 (*NEU3*), pyruvate dehydrogenase kinase 4 (*PDK4*), fatty acid transport protein 4 (*SLC27A4*), thiamine transporter 1 (SLC19A2), NAD kinase (*NADK*), BTB domain and CNC homolog 2 (*BACH2*) and ARID domain-containing protein 5B (*ARID5B*). We have also compared the results based on *K* estimates with the sets of hub genes forming part of the co-expression modules generated with the WGCNA algorithm [38]. By doing so, we found several genes that in both approaches were identified as top central players in the metabolic response to food intake. For instance, *BACH1* and *CREB1* genes were among the top hubs in the Blue co-expression module corresponding to the AL-T0/AL-T1 contrast (Additional file 9: Table S9). With respect to AL-T0/ALT2, *SCAMP2*, *NEU3* and *PDK4* genes within the Green co-expression module were also among the top hub transcripts, whereas *BACH2* and *ARID5B* occupied intermediate positions in the ranking of hub genes (Additional file 9: Table S9).

Additionally, we used the TargetScan algorithm to evaluate the accuracy of the miRNA-to-mRNA interactions predicted with PCIT and 3′-UTR seed matching. Four highly expressed DE miRNA*s (*ssc-miR-148a-3p, ssc-miR-1, ssc-miR-493-5p and ssc-let-7/ssc-miR-98) were selected for this task. From a total of 30 different mRNA genes predicted to be targets of the selected miRNAs (Additional file 6: Table S6), 14 showed conserved and putatively valid interactions (context++ score > 75% percentile) according to predictions made with the TargetScan algorithm (Additional file 10: Table S10)*.*

Particularly interesting was the case of the miRNAs predicted to bind the 3′-UTR sequence of the *PDK4* mRNA (Additional file 11: Table S11), which happened to be the most highly downregulated gene in the AL-T0/AL-T2 contrast (Additional file 2: Table S2). Among the 7 predicted miRNAs with putative 7mer-m8 binding sites in the *PDK4* 3′-UTR, only two sites appeared to be consistently conserved when compared against the corresponding orthologous regions in other phylogenetically related species (Additional file 15: Figure S3, Additional file 10: Table S10). Noteworthy, the two conserved sites are predicted to bind to ssc-miR-148a-3p and ssc-miR-493-5p, which were two of the most highly DE miRNAs in the AL-T0/AL-T2 contrast (Table 1).

Besides, after estimating the RIF score for each co-expressed miRNA, results were ranked according to their regulatory relevance. A complete list of all RIF values for miRNAs is presented in Additional file 12: Table S12. Moreover, a list of the top 5 ranking positive and negative regulatory miRNAs according to their RIF1 and RIF2 scores is presented in Tables 3 and 4, respectively. Interestingly, we observed a high correspondence between miRNAs classified as DE with the *edgeR* tool and miRNAs categorized by the PCIT and RIF algorithms as meaningful regulators (Tables 1, 3 and 4, Additional files 6 and 12: Table S6 and S12). For instance, ssc-miR-32, which was DE in the two considered contrasts, ranked as the second (AL-T0/AL-T1) and third (AL-T0/AL-T2) most relevant miRNA in terms of RIF1 (Table 3**,** Additional file 12: Table S12). The DE miRNAs (AL-T0/AL-T2) ssc-miR-339 and ssc-miR-1 were also detected as relevant in terms of RIF1 score (Table 3). When considering RIF2 and AL-T0/AL-T2, the ssc-miR-1285, ssc-miR-129a-3p, ssc-miR-296-5p, ssc-miR-374a-3p and ssc-miR-7-5p DE miRNAs happened to be among the top predicted regulators (Table 4). In the AL-T0/AL-T2 contrast, several additional DE miRNAs also belonged to the group of the top 10 most relevant regulators according to their RIF scores, e.g. ssc-miR-22-3p for RIF1 and ssc-miR-148a-3p or ssc-miR-493-5p for RIF2 (Additional file 12: Table S12).

**Table 3:** Top five positive and negative regulatory microRNAs according to their Regulatory Impact Factor 1 (RIF1)

Contrast	RIF1
AL-T0/AL-T1^a^	
ssc-miR-450b-5p	1.7939
ssc-miR-32	1.7041
ssc-miR-136-5p	1.3928
ssc-miR-542-3p	1.2969
ssc-miR-19a	1.2620
ssc-miR-339-3p	−0.9864
ssc-miR-421-5p	−0.9871
ssc-miR-503	−1.1680
ssc-miR-326	−1.2569
ssc-miR-128	−1.2830
AL-T0/AL-T2^a^	
ssc-miR-9858-5p	2.7536
ssc-miR-148b-5p	2.4587
ssc-miR-32	2.3825
ssc-miR-129a-5p	1.9010
ssc-miR-7139-5p	1.3797
ssc-let-7g	−1.0629
ssc-miR-130b-5p	−1.1300
ssc-miR-339	−1.2069
ssc-miR-1	−1.2630
ssc-miR-326	−1.3955

^a^AL-T0: Duroc gilts in a fasting condition (*N* = 11); AL-T1: Duroc gilts slaughtered after 5 h of food intake (*N* = 12); AL-T2: Duroc gilts slaughtered after 7 h of food intake (*N* = 12)

**Table 4:** Top five positive and negative regulatory microRNAs according to their Regulatory Impact Factor 2 (RIF2)

Contrast	RIF2
AL-T0/AL-T1^a^	
ssc-miR-129a-3p	1.8373
ssc-miR-219a	1.4996
ssc-miR-128	1.4256
ssc-miR-503	1.2053
ssc-miR-450b-3p	1.0913
ssc-miR-455-5p	−0.9408
ssc-miR-296-5p	−1.0613
ssc-miR-143-3p	−1.3923
ssc-miR-542-3p	−1.4893
ssc-miR-450b-5p	−1.5585
AL-T0/AL-T2^a^	
ssc-miR-1285	2.2089
ssc-miR-206	1.7993
ssc-let-7d-5p	1.7537
ssc-miR-129a-3p	1.5109
ssc-miR-129a-5p	1.3630
ssc-miR-296-5p	−1.6368
ssc-miR-374a-3p	−1.6758
ssc-miR-148b-5p	−1.8280
ssc-miR-7-5p	−2.0613
ssc-miR-7139-5p	−2.6767

^a^AL-T0: Duroc gilts in a fasting condition (*N* = 11); AL-T1: Duroc gilts slaughtered after 5 h of food intake (*N* = 12); AL-T2: Duroc gilts slaughtered after 7 h of food intake (*N* = 12)

### Relationship between weighted gene co-expression modules and meat quality and muscle fatty acids composition traits

The WGCNA algorithm applied to mRNA and miRNA expression estimates in the AL-T0/AL-T1 and AL-T0/AL-T2 matrices made possible the identification of 5 and 10 gene co-expression modules, respectively (Additional file 15: Figure S4 and S5), excluding miRNA-only co-expression modules. Among these, the identified modules for the AL-T0/AL-T1 contrast were significantly associated with the following meat quality and fatty acids composition phenotypes measured in the *gluteus medius* muscle: meat lightness (L*), intramuscular pH (PHGM), intramuscular fat content (GMIMF), palmitic acid content (C16:0), linoleic acid content (C18:2-ω6), arachidonic acid content (C20:4), omega-6 fatty acids content (ω6), omega-6/omega-3 ratio (ω6/ω3), polyunsaturated fatty acids content (PUFA) and polyunsaturated/saturated fatty acids ratio (PUFA/SFA), as shown in Additional file 13: Table S13. Regarding the AL-T0/AL-T2 contrast, *gluteus medius* phenotypes showing significant associations with co-expression modules were: meat redness (a*), pH measured 45 min post-mortem (PH45GM), linoleic acid content (C18:2-ω6), arachidonic acid content (C20:4), omega-3 (ω3), omega-6/omega-3 ratio (ω6/ω3), unsaturated fatty acids content (UFA) and polyunsaturated/saturated fatty acids ratio (PUFA/SFA) and saturated/unsaturated fatty acids ratio (SFA/UFA) (Additional file 14: Table S14). A detailed list of all analyzed phenotypes is shown in Additional file 1: Table S1. *P*-values measuring the significance of the contribution of each gene within co-expression modules to significantly correlated phenotypic traits can be found in Additional files 13: Table S13 (AL-T0/AL-T1) and 14: Table S14 (AL-T0/AL-T2).

## Discussion

### Coding and non-coding RNAs show highly divergent patterns of expression in the porcine muscle

By comparing mRNAs, miRNAs and lincRNAs expression patterns, we have observed that the expression of mRNAs in the porcine skeletal muscle is, on average, substantially higher than that of miRNAs and lincRNAs (Fig. 2). This finding was expected because previous studies in humans have reflected the same trend for lincRNAs [42, 43] and miRNAs [44]. On the other hand, we have also observed an inverse relationship between the expression means of mRNA and lincRNA genes and the magnitude of BCVs (Fig. 3a, c), whereas such trend was not obvious for miRNAs (Fig. 3b).

With regard to differential dispersion, the number of DD mRNA and miRNA genes was much lower than that of DE mRNA and miRNA genes, indicating that nutrient supply has a stronger impact on the mean expression of genes rather than on their BCV. Of course, these two parameters are closely related, so decreases in the mean expression of genes are usually accompanied by increases in the variance of expression (and vice versa), being such trend particularly true for mRNAs and lincRNAs. In contrast, miRNAs showed a very resilient and stable pattern of expression across replicates (Figs. 3b and 4b).

While nutrient supply induced substantial changes in the expression of mRNAs (Additional file 2: Table S2), the absolute number of DE miRNAs was much lower (Table 1), whereas no DE lincRNAs were detected. This result is probably not due to a limited accuracy of RNA-Seq in detecting differential gene expression, because previous experiments [40] showed a high consistency between differential gene expression results obtained with RNA-Seq and real time quantitative PCR data in the same experimental system. However, it should be taken into account that the absolute numbers of annotated porcine miRNAs and lincRNAs are much smaller than those of mRNAs. Indeed, when the number of DE genes is expressed as a proportion (i.e. number of DE genes/number of total analyzed expressed genes), the total amount of DE mRNAs happened to be 1.39% (AL-T0/AL-T1) and 4.06% (AL-T0/AL-T2). In the case of miRNAs, such proportions were 2.09% (AL-T0/AL-T1) and 9.79% (AL-T0/AL-T2). Moreover, the average |FC| of DE mRNAs was 2.12-fold and 2.02-fold in AL-T0/AL-T1 and AL-T0/AL-T2 respectively, while for miRNAs, changes of 1.9-fold (AL-T0/AL-T1) and 1.85-fold (AL-T0/AL-T2) were detected. In the light of these results, it should be concluded that both mRNAs and miRNAs show consistent patterns of differential expression in response to food intake, while no conclusive evidence has been obtained for lincRNAs. This latter observation could be due to the poor annotation of lincRNAs as well as to their low expression levels and elevated within group expression variability (Figs. 2 and 3c), which ultimately would make the differential expression analysis much less powerful to detect significant differences.

Nevertheless, the high variance in the expression of lincRNAs contrasted strongly with the stable patterns of expression across contrasts displayed by miRNAs (Figs. 2 and 3b, c). This high stability might be due to the fact that the expression and silencing activity of miRNAs are decoupled to some extent [36]. There are several factors that explain such circumstance. For instance, miRNAs can be sequestered by pseudogene, mRNA, lincRNA or circular RNA transcripts with repeated miRNA antisense sequences (the so-called miRNA sponges), thus limiting their availability to regulate the expression of target RNAs [45–47]. Moreover, compelling evidence has been accumulating during past years highlighting the exceptional stability of certain miRNAs, which show half-lives of days [48, 49]. This long half-life might be explained by the protective effect of the Argonaute protein in isolating naked single-stranded small miRNA molecules from exonucleases within the cell environment [50]. Besides, miRNAs might localize to cell compartments other than the cytosol, where they exert functions unrelated with the modulation of mRNA levels [51]. Last but not least, the expression levels of miRNAs do not necessarily correlate with their functional availability as a part of the RNA-induced silencing complex [36].

### Differentially expressed and dispersed miRNAs are related with the regulation of key metabolic processes in the skeletal muscle

As shown in Tables 1 and 2, several miRNAs were detected as either being DE and/or DD in the AL-T0/AL-T1 and AL-T0/AL-T2 contrasts. Among the DE miRNAs, we found that ssc-miR-1 and ssc-miR-148a were two of the most expressed and DE miRNAs in AL-T0/AL-T1 and AL-T0/AL-T2 contrasts (Table 1), whereas ssc-miR-7-5p was the most highly differentially upregulated miRNA in AL-T1 gilts. Both miR-7 and miR-1 regulate the mTOR-related cell response to nutrient availability. For instance, miR-1 was found to be directly upregulated by the myogenic differentiation 1 (*MYOD1*) gene [52], which is a transcription factor essential for skeletal muscle development and myocyte fusion [53] and also functions as a circadian modulator in the peripheral muscle clock [54]. Noteworthy, *MYOD1* was also significantly upregulated in the AL-T0/AL-T2 contrast (Additional file 2: Table S2), a finding that agrees well with the observed upregulation of ssc-miR-1 (Table 1). Additionally, miR-7 has been also associated with the Akt-mTOR and PI3K/Akt signaling by targeting the insulin receptor substrate 2 (*IRS2*) and the phosphoinositide 3-kinase catalytic subunit δ (*PIK3CD*) [55, 56], two genes that are integrated in the coordinated signaling cascade in response to nutrient supply to promote skeletal muscle growth and differentiation.

Regarding the miR-148 family, it has been reported that these miRNAs play a key role in cholesterol metabolism [57–59] and insulin homeostasis [60]. In a fasting/feeding study resembling ours, Goedeke et al. [59] reported that miR-148a binds the 3′-UTR of the low density lipoprotein receptor (*LDLR*) mRNA leading to the accumulation of low-density lipoprotein (LDL) cholesterol in blood plasma. Similar results were reported by Rotllan et al. [61]. Furthermore, Goedeke et al. [59] suggested that the sterol regulatory element-binding transcription factor 1 (*SREBF1*) may activate the expression of miR-148a by targeting conserved E-box motifs in the miRNA promoter. In the same study, the role of the ATP-binding cassette 1 (*ABCA1*) gene in the regulation of high-density lipoprotein (HDL) cholesterol levels was explored, and a binding site for miR-148a in the 3′-UTR of *ABCA1* transcripts was predicted, thus providing a functional explanation for the inhibitory effect of miR-148a on plasma HDL cholesterol levels [59]. Other studies have also linked miRNAs belonging to the miR-148 family with angiogenesis and glucose metabolism through insulin like growth factor 1 receptor (*IGF1R*) target inhibition [62].

With respect to other relevant DE miRNAs detected in our study, the miR-30 family and miR-503 have been described to be involved in skeletal muscle differentiation and fiber-type composition [63, 64]. Moreover, they also regulate adipogenesis [65], a role that has also been reported for miR-148a [66] and miR-22 [67]. Furthermore, the observed downregulation of miR-22 after food ingestion (Table 1) could be the consequence of the active influx of glucose within muscle cells after nutrient supply. Indeed, the glucose transporter 1 (*GLUT1*) mRNA is targeted by miR-22 [68]*.* A similar reasoning could be extended to miR-17-5p, which binds to the glucose transporter 4 (*GLUT4*) mRNA [69] and that was DD but not DE after feed intake (Table 2).

### Relevant miRNA-to-mRNA regulatory interactions in response to nutrient supply

Co-expression network analyses highlighted that the majority of DE miRNAs were also potentially meaningful regulatory factors (Tables 1, 3 and 4, Additional file 12: Table S12). Other miRNAs also emerged as potential regulators (Tables 3 and 4, Additional file 12: Table S12) despite not being detected as significantly DE, a finding that would be in agreement with the very stable and low expression levels detected for most miRNAs (Figs. 2 and 3b). These results evidence the interest of reconstructing regulatory networks in order to gain new biological insights that canonical differential expression analysis cannot yield [70]. Several critical downregulated transcription factors in AL-T1 animals were identified as potential co-expressed targets of ssc-miR-1 and ssc-miR-148a-3p DE miRNAs (Additional files 2 and 6: Table S2 and Table S6), e.g. the myogenic factor 6 (*MYF6*), FOS-related antigen 2 (*FOSL2*) and arrestin domain-containing protein 3 (*ARRDC3*) for ssc-miR-1, and thioredoxin interacting protein (*TXNIP*) and fasting-induced gene protein (*DEPP1*) for ssc-miR-148a. The *MYF6* gene has been previously associated with the regulation of myogenesis and skeletal muscle cell differentiation [8, 71]. A proliferation modulating function has also been described for *TXNIP* [72] as well as for *FOSL2* [73], which is also involved in leptin expression regulation [74], whereas *DEPP1* downregulation has been associated with autophagy inhibition [75]. Moreover, ssc-miR-32 and ssc-miR-7-5p, two miRNAs that were differentially upregulated in AL-T1 gilts (Table 1), were predicted to target several relevant genes (Additional file 6: Table S6) such as the activating transcription factor 3 (*ATF3*), a key regulator of glucose and energy metabolism [76, 77] which was significantly downregulated in both AL-T0/AL-T1 and AL-T0/AL-T2 contrasts (Additional file 2: Table S2). Other relevant additional transcripts that formed part of the miRNA-to-mRNA interconnected networks were, to mention a few, the Kruppel-like factor 15 (*KLF15*), early growth factor 1 (*EGR1*) and ARID domain-containing protein 5B (*ARID5B*), all of which play key roles in muscle lipid metabolism [8, 78, 79], or myogenin (*MYOG*), a gene that is crucial for muscle development and differentiation [80].

With regard to AL-T2 gilts, it is worth mentioning the *PDK4* gene, which happened to be the most extremely downregulated mRNA transcript (Additional file 2: Table S2) and was also detected as DD in the AL-T0/AL-T2 contrast (Additional file 3: Table S3). After reconstructing meaningful miRNA-to-mRNA interactions, seven miRNAs (ssc-miR-148a-3p, ssc-miR-151-3p, ssc-miR-30a-3p, ssc-miR-30e-3p, ssc-miR-421-5p, ssc-miR-493-5p and ssc-miR-503) were predicted to have putative binding sites in the *PDK4* 3′-UTR (Additional files 6 and 11: Table S6 and Table S11). Noteworthy, all these miRNAs were significantly upregulated in the skeletal muscle of AL-T2 gilts (Table 1), with the only exception of ssc-miR-503, (Table 1). Our findings agree well with a cooperative and synergistic interaction between the aforementioned miRNAs and the *PDK4* mRNA, that would result in its strong downregulation observed in AL-T2 pigs (Additional file 2: Table S2). Interestingly, among the set of miRNAs significantly co-expressed with *PDK4* mRNAs, and also predicted to interact with its 3′-UTR, ssc-miR-148a-3p and ssc-miR-493-5p were two of the most significantly upregulated miRNAs in AL-T2 gilts (Table 1). Moreover, the TargetScan analysis [35] showed that both miRNAs have evolutionarily conserved binding sites in the 3′-UTR of the *PDK4* gene (Additional file 15: Figure S3, Additional file 10: Table S10). We may hypothesize that ssc-miR-148a-3p and ssc-miR-493-5p play a key role in the downregulation of the *PDK4* mRNA after food intake, but such hypothesis still needs experimental verification.

**Fig. 6:**
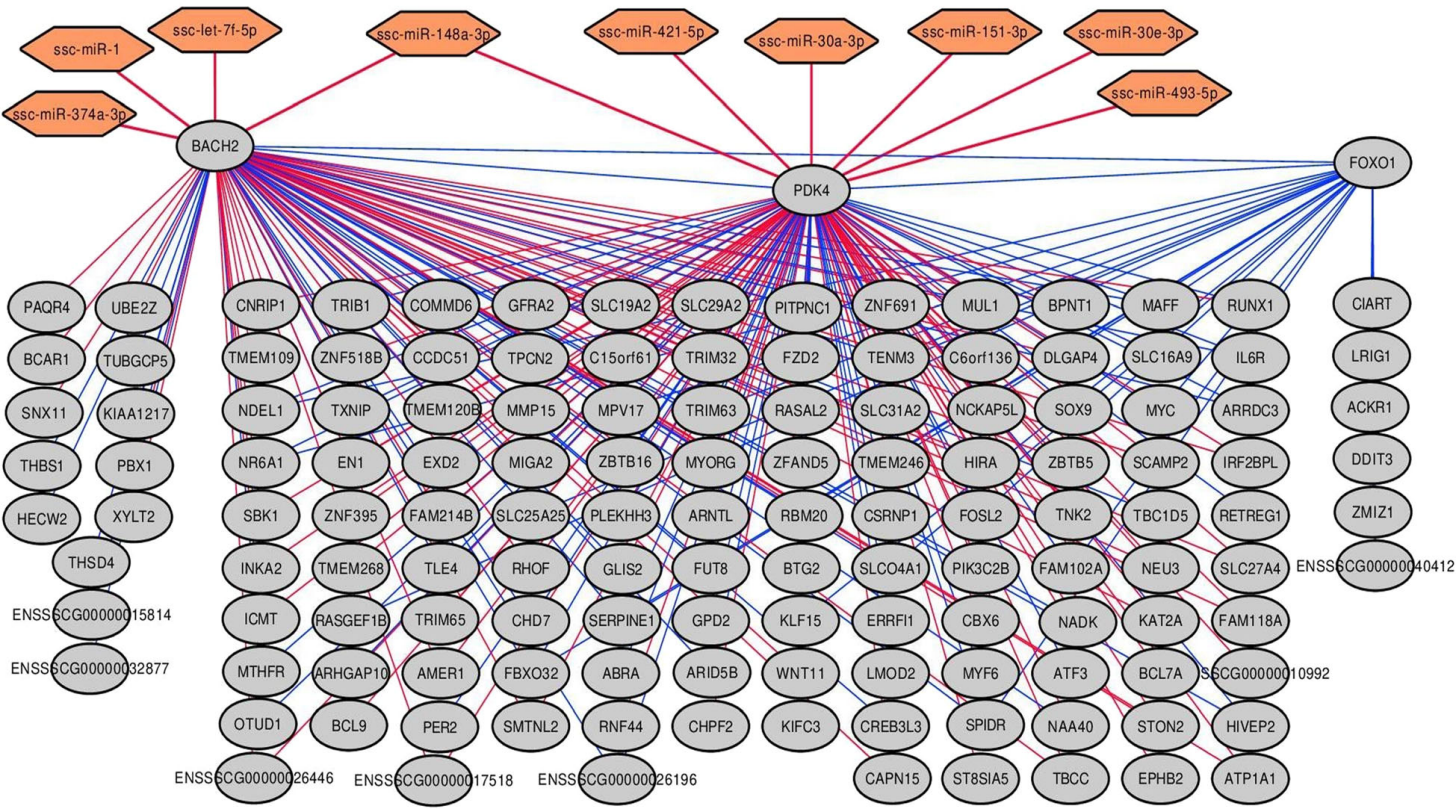
Selected miRNA-to-mRNA and mRNA-to-mRNA co-expression network according to the PCIT algorithm in the AL-T0/AL-T2 contrast. Differentially expressed miRNAs and mRNAs were considered. Only significant correlations below − 0.5 for miRNA-to-mRNA and above |0.7| for mRNA-to-mRNA interactions where selected. Red and blue edges indicate negative and positive correlations in the co-expression network, respectively

Co-expression network analysis also indicated that the *PDK4* gene might interact with a broad array of mRNA transcripts (Fig. 6). Among these, several have been already mentioned (*MYF6, FOSL2, KLF15, ARID5B, DEPP1*, *MYOG* or *TXNIP*) while others have not, e.g. aryl hydrocarbon receptor nuclear translocator like (*ARNTL*), forkhead box O1 (*FOXO1*), neuronal PAS domain protein 2 (*NPAS2*), BTB domain and CNC homolog 2 (*BACH2*) or the period circadian regulator 2 (*PER2*). The *PDK4* gene is one of the master regulators of glucose and lipid metabolism [81]. Moreover, the PDK4 protein is located in the matrix of the mitochondria and inhibits the pyruvate dehydrogenase complex, which catalyzes the conversion of pyruvate to acetyl-CoA, and hence it is responsible of the decrease in glucose utilization and the upregulation of fatty acid oxidation in energy-deprived cells under fasting conditions [82, 83].

The observed coordinated downregulation of both *PDK4* and *FOXO1* mRNAs in the AL-T0/AL-T2 contrast (Additional file 2: Table S2) is consistent with the active energy production and fatty acid synthesis of muscle cells in response to nutrient supply, as already reported by Cardoso et al. [5]. In fact, the activation of *FOXO1* is known to enhance *PDK4* transcription by binding to its promoter region [84, 85]. Besides, the *BACH2* transcription factor was also predicted to be regulated by ssc-miR-148a-3p (Additional files 6 and 10: Table S6 and Table S10) as well as to interact with both *FOXO1* and *PDK4* mRNAs **(**Fig. 6). These findings agree well with the previously described role of *BACH2* as a transcriptional activator of *FOXO1* by binding to its promoter region [86–88]. The presence of genes involved in the maintenance of circadian rhythms (*NPAS2*, *ARNTL* and *PER2*) was also relevant, as the expression of the *PDK4* mRNA is subjected to circadian fluctuations in response to light shifting and insulin and fatty acids availability [89–91]. Noteworthy, the potential implications of nutrition in the regulation of the porcine peripheral clocks was already discussed in two previous studies using the very same animal material and experimental design reported herewith [5, 40], a result that would be in agreement with the reconstructed *PDK4* miRNA-to-mRNA interaction network reported in this study.

### mRNA-to-mRNA hub genes reveal glucose and lipid metabolism changes induced by food intake

Hub scoring of meaningful mRNA genes from selected co-expression interaction networks also allowed the identification of several relevant transcripts involved in organizing the cell response to nutrient availability (Additional file 8: Table S8), and several of these were also detected as hub genes in WGCNA analyses (Additional file 9: Table S9). With respect to AL-T0/AL-T1, the *NR1D2* gene was the most prominent hub gene among all other transcripts, despite the fact that it was not detected as DE. This transcription factor and its paralog Rev-Erbα (*NR1D1*) contribute to establish links between circadian rhythms and cell metabolism regulation [92]. Remarkably, other relevant top hub genes were not DE, e.g. the *BACH1* transcription factor, whose inhibition has been associated with an increased protection against oxidative stress [93], *ETS1*, which mediates *FOXO1* acetylation and regulates gluconeogenesis in fasting-feeding cycles [94] or *CREB1*, an important cofactor for the peroxisome proliferator-activated receptor γ coactivator 1-α (*PPARGC1A*), a gene that plays a key role in insulin-mediated glucose uptake [95].

Regarding hub genes detected in the AL-T0/AL-T2 contrast (Additional file 8: Table S8), *SCAMP2* has been related to glucose transporters trafficking during insulin stimulation [96], whereas *NEU3*, which was also highly upregulated in fed gilts (Additional file 2: Table S2), stimulates insulin sensitivity and glucose tolerance [97]. Other relevant examples are: *SLC27A4*, responsible for long chain fatty acids metabolism and trafficking [98], *SLC19A2*, also highly downregulated in fed gilts (Additional file 2: Table S2) and reported as being negatively regulated by glucose uptake [99], and NADK, a protein that phosphorylates NAD^+^ to generate NADP^+^, a metabolite tightly linked with the regulation of circadian rhythms [100].

These findings agree well with data previously reported by Cardoso et al. [5], as well as with enrichment analyses described in this study (Additional files 4 and 5: Table S4 and Table S5), where many DE genes associated with diverse glucose and lipid metabolism pathways and GO terms were highlighted. Other biological processes like muscle proliferation associated to nutrient availability and circadian regulation provided compelling evidence about the complex machinery triggered in the skeletal muscle to respond to nutrient supply after food ingestion.

### Weighted co-expression analyses revealed hub genes related with lipids metabolism regulation

Among the gene co-expression modules detected with the WGCNA approach [38], the so-called Red and Purple clusters (Additional file 14: Table S14), corresponding to the AL-T0/AL-T2 contrast, contained several relevant lipid metabolism-related genes such as the fatty acid binding protein 4 (*FABP4*), carbohydrate-responsive element-binding protein (*MLXIPL*), fatty acid synthase (*FASN*), thyroid hormone responsive protein (*THRSP*), stearoyl-CoA desaturase (*SCD*), acetyl-CoA carboxylase 1 (*ACACA*) or the secreted frizzled-related proteins 1 and 5 (*SFRP1* and *SFRP5*), as well as other loci such as the cholinergic receptor nicotinic δ subunit (*CHRND*). From these, the *MLXIPL*, *FASN*, *SCD*, *SFRP1*, *SFRP5* and *THRSP* genes were also significantly upregulated in AL-T2 gilts after feeding (Additional file 2: Table S2).

Interestingly, the active/non-active conformation of the muscle acetylcholine receptor function regulating motor nerve-muscle communication and muscle contraction is tightly associated with the concentration of certain surrounding fatty acid components, contributing to stabilize or destabilize its functionality [101], a phenomenon that could explain the observed association between its δ subunit (*CHRND*) and the content of ω-3 fatty acids and ω6/ω3 content ratio in the *gluteus medius,* as shown in Additional file 14: Table S14.

Other genes that are key regulators of lipid metabolism such as *SCD*, *ACACA*, *FABP4, SFRP1, THRSP* or the hub genes *SFRP5* and *FASN* (Additional file 9: Table S9), also clustered in a tight co-expression module and they were significantly associated with linoleic and arachidonic fatty acids content in the *gluteus medius* muscle (Additional file 14: Table S14). The SFRP5 protein has been thoroughly studied as a central regulator of lipid accumulation and adipocytes differentiation, which are a result of an increased mitochondrial respiration promoted by SFRP5 blocking of Wnt signaling, hence repressing Wnt-induced oxidative metabolism [102]. The other identified SFRP element (*SFRP1*) has also been reported to be located in a genomic region overlapping a QTL for meat marbling [103, 104]. Moreover, the *THRSP, MLXIPL* and *FASN* upregulation detected in our analyses (Additional file 2: Table S2), as well as their contribution to intramuscular lipid content (Additional files 9 and 14: Table S9 and Table S14) could be a reflection of the intramuscular adipocyte proliferation triggered by the nutrient supply provided to AL-T2 fed gilts [105]. Indeed, the *MLXIPL* is a key carbohydrate-signaling transcription factor whose activity is enhanced by glucose metabolites, thus binding to carbohydrate response elements (ChoREs) present in the promoters of several key lipid genes such as *FASN* [106].

## Conclusions

In conclusion, we have demonstrated that the profiles of expression of lincRNAs and miRNAs in the *gluteus medius* muscle of pigs are very different than those observed for mRNAs. For instance, the mean and the variance of gene expression are closely interdependent parameters in the case of mRNAs, while miRNAs do not show such trend. We have also demonstrated that feeding induces changes mainly in the mean expression of genes rather than on their expression variance, a parameter which remains relatively unaffected by nutrient supply. Finally, co-expression network analyses predict that miRNAs and hub mRNA genes may play an essential role in the regulation of mRNAs showing differential expression upon feeding. Such regulatory interactions predicted with in silico tools should be validated experimentally in order to verify their occurrence as well as to infer their biological significance in the context of porcine muscle metabolism and nutrition.

## Supplementary information

**Additional file 1:** **Table S1.** Meat quality and *gluteus medius* fatty acids composition traits recorded in AL-T0, AL-T1 and AL-T2 Duroc gilts.

**Additional file 2:** **Table S2.** Differentially expressed mRNAs in the AL-T0/AL-T1 and AL-T0/AL-T2 contrasts.

**Additional file 3:** **Table S3.** Differentially dispersed mRNAs in the AL-T0/AL-T1 and AL-T0/AL-T2 contrasts.

**Additional file 4:** **Table S4.** Pathway Enrichment and GO Enrichment analyses in the AL-T0/AL-T1 contrast.

**Additional file 5:** **Table S5.** Pathway Enrichment and GO Enrichment analyses in the AL-T0/AL-T2 contrast.

**Additional file 6:** **Table S6.** miRNA-to-mRNA significant interactions detected with the PCIT algorithm in the AL-T0/AL-T1 and AL-T0/AL-T2 contrasts.

**Additional file 7:** **Table S7.** mRNA-to-mRNA significant interactions detected with the PCIT algorithm in the AL-T0/AL-T1 and AL-T0/AL-T2 contrasts.

**Additional file 8:** **Table S8.** Estimated Hub scores (*K*) values for genes differentially expressed in the AL-T0/AL-T1 and AL-T0/AL-T2 contrasts.

**Additional file 9:** **Table S9.** Scaled Kleinberg’s hub scores per gene for each WGCNA module significantly correlated with phenotypic traits in the AL-T0/AL-T1 and AL-T0/AL-T2 contrasts.

**Additional file 10:** **Table S10.** Identification of conserved mRNA targets for selected highly expressed and differentially expressed miRNAs based on the TargetScan Context++ score.

**Additional file 11:** **Table S11.** miRNAs predicted to bind the 3′-UTR of the porcine *PDK4* gene.

**Additional file 12:** **Table S12.** Regulatory Impact Factor scores (RIF1 and RIF2) for miRNAs classified by the PCIT algorithm as meaningful regulators in the AL-T0/AL-T1 and AL-T0/AL-T2 contrasts.

**Additional file 13:** **Table S13.** Gene co-expression modules significantly associated with meat quality and fatty acids composition traits according to the WGCNA algorithm (AL-T0/AL-T1 contrast).

**Additional file 14:** **Table S14.** Gene co-expression modules significantly associated with meat quality and fatty acids composition traits according to the WGCNA algorithm (AL-T0/AL-T2 contrast).

**Additional file 15:** **Figure S1.** Sequencing depth obtained for samples analyzed in each one of the two contrasts (AL-T0/AL-T1 and AL-T0/AL-T2). **Figure S2.** Joint Principal Component Analysis (PCA) clustering of *gluteus medius* skeletal muscle samples (11 AL-T0, 12 AL-T1 and 12 AL-T2 samples) according to the expression profiles of **(A)** mRNAs, **(B)** microRNAs and **(C)** lincRNAs. **Figure S3.** Phylogenetically conserved 7mer-8 m predicted binding sites in the 3′- UTR of the pig *PDK4* gene for **(A)** ssc-miR-148a-3p and **(B)** ssc-miR-493-5p porcine miRNAs. The TargetScan software was used for generating conservation graphs across the investigated mammalian species. Red nucleotides show complementary matching base-pairs between the seed of the mature miRNA and the 3’UTR of the pig *PDK4* gene. **Figure S4.** Gene co-expression module association with meat quality and fatty acids composition traits in the AL-T0/AL-T1 contrast as determined with the WGCNA tool. **Figure S5.** Gene co-expression module association with meat quality and fatty acids composition traits in the AL-T0/AL-T2 contrast as determined with the WGCNA tool.

## Data Availability

The small RNA-Seq data set used and/or analyzed in the current study is available at the Sequence Read Archive (SRA) database (BioProject: PRJNA595998). The previously published RNA-Seq data set was also submitted to the SRA database (BioProject: PRJNA386796).

## Abbreviations

BCV: Biological Coefficient of Variation
CPM: Counts-Per-Million
CV: Coefficient of Variation
DD: Differentially Dispersed
DE: Differentially Expressed
FC: Fold Change
GEV: Gene Expression Variability
GO: Gene Ontology
GS: Gene Significance
K: Hub Score
lincRNA: long intergenic non-coding RNA
MEs: Module Eigengenes
miRNA: microRNA
mRNA: messenger RNA
PCA: Principal Component Analysis
PCIT: Partial Correlation with Information Theory
PIF: Phenotype Impact Factor
*r*: Pearson Correlation coefficient
RIF: Regulatory Impact Factor
Rlog: Regularized log_2_
TOM: Topological Overlapping matrix
WGCNA: Weighted Gene Correlation Network Analysis
