# Supplementary material for: Co-expression network analysis predicts a key role of microRNAs in the adaptation of the porcine skeletal muscle to nutrient supply
Source: J Anim Sci Biotechnol. 2020 Jan 17;11:10. doi: 10.1186/s40104-019-0412-z (PMC6966835; doi:10.1186/s40104-019-0412-z)

Co-expression network analysis predicts a key role of microRNAs in the adaptation of the porcine skeletal muscle to nutrient supply

Emilio Mármol-Sánchez[1], Yuliaxis Ramayo-Caldas[2], Raquel Quintanilla[2], Tainã Figueiredo Cardoso[1]*, Rayner González-Prendes[3], Joan Tibau[2], Marcel Amills[1,4]

[1]Centre for Research in Agricultural Genomics (CRAG), CSIC-IRTA-UAB-UB, Universitat Autònoma de Barcelona, 08193 Bellaterra, Spain.

[2]Animal Breeding and Genetics Program, Institute for Research and Technology in Food and Agriculture (IRTA), Torre Marimon, 08140 Caldes de Montbui, Spain.

[3]Universitat de Lleida - Agrotecnio Center, Department of Animal Science, 25198, Lleida, Spain.

[4]Departament de Ciència Animal i dels Aliments, Universitat Autònoma de Barcelona, 08193 Bellaterra, Barcelona, Spain.

ADDITIONAL FIGURES:

Additional Fig. 1: Sequencing depth obtained for samples analyzed in each one of the two contrasts (AL-T0/AL-T1 and AL-T0/AL-T2).

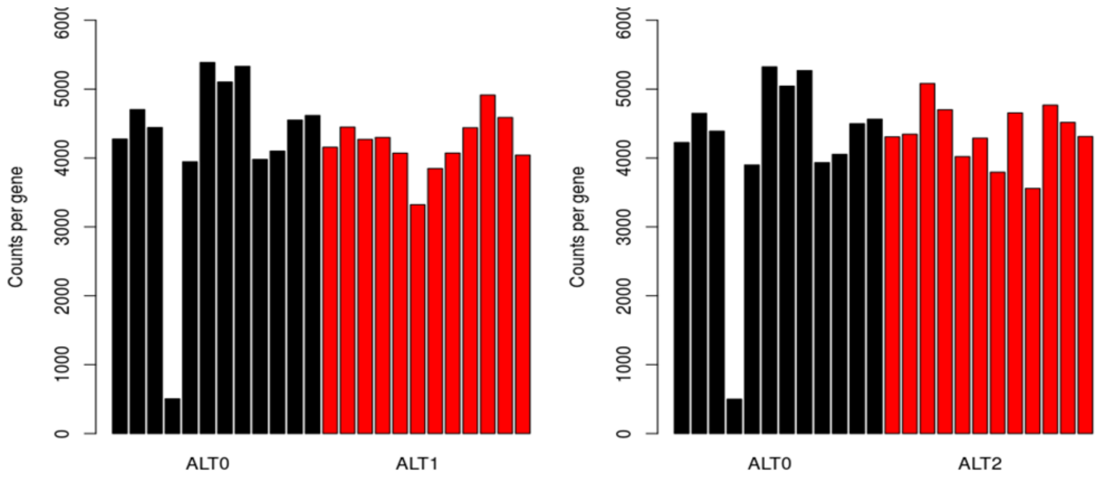

Additional Fig. 2: Joint Principal Component Analysis (PCA) clustering of *gluteus medius* skeletal muscle samples (11 AL-T0, 12 AL-T1 and 12 AL-T2 samples) according to the expression profiles of **(A)** mRNAs, **(B)** microRNAs and **(C)** lincRNAs.

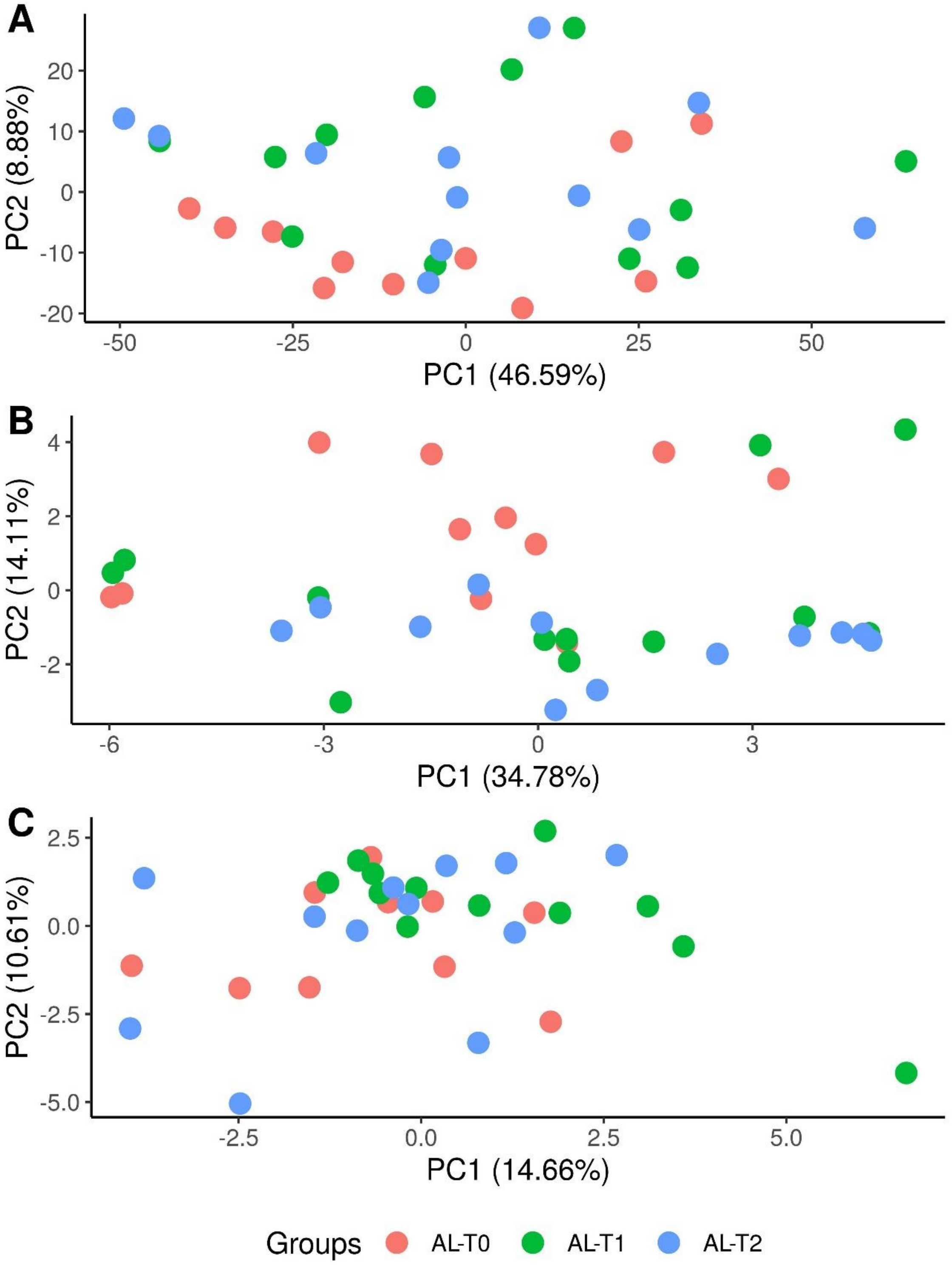

Additional Fig. 3: Phylogenetically conserved 7mer-8m predicted binding sites in the 3’- UTR of the pig *PDK4* gene for **(A)** ssc-miR-148a-3p and **(B)** ssc-miR-493-5p porcine miRNAs. The TargetScan software was used for generating conservation graphs across the investigated mammalian species. Red nucleotides show complementary matching base-pairs between the seed of the mature miRNA and the 3’UTR of the pig *PDK4* gene.

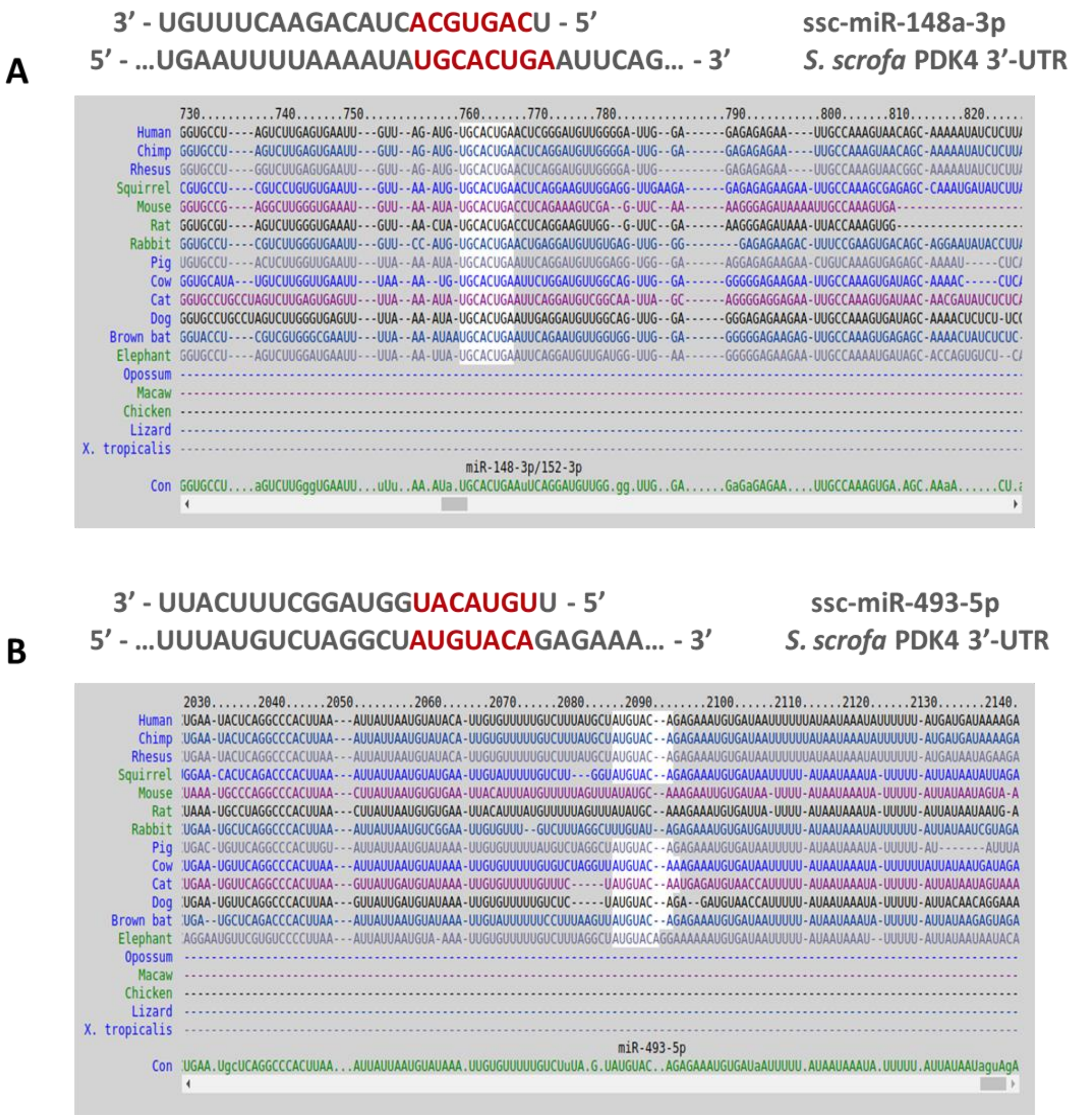

Additional Fig. 4: Gene co-expression module association with meat quality and fatty acids composition traits in the AL-T0/AL-T1 contrast as determined with the WGCNA tool.

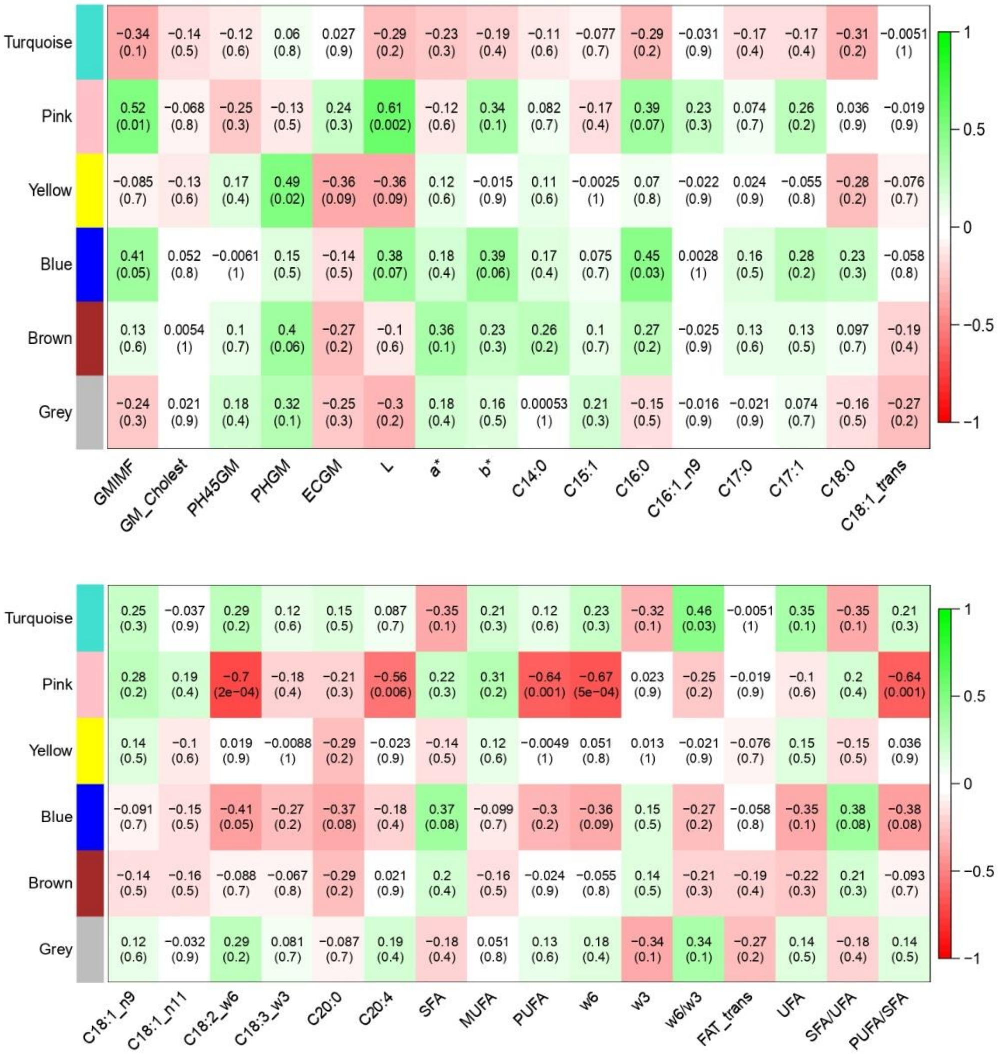

Additional Fig. 5: Gene co-expression module association with meat quality and fatty acids composition traits in the AL-T0/AL-T2 contrast as determined with the WGCNA tool.

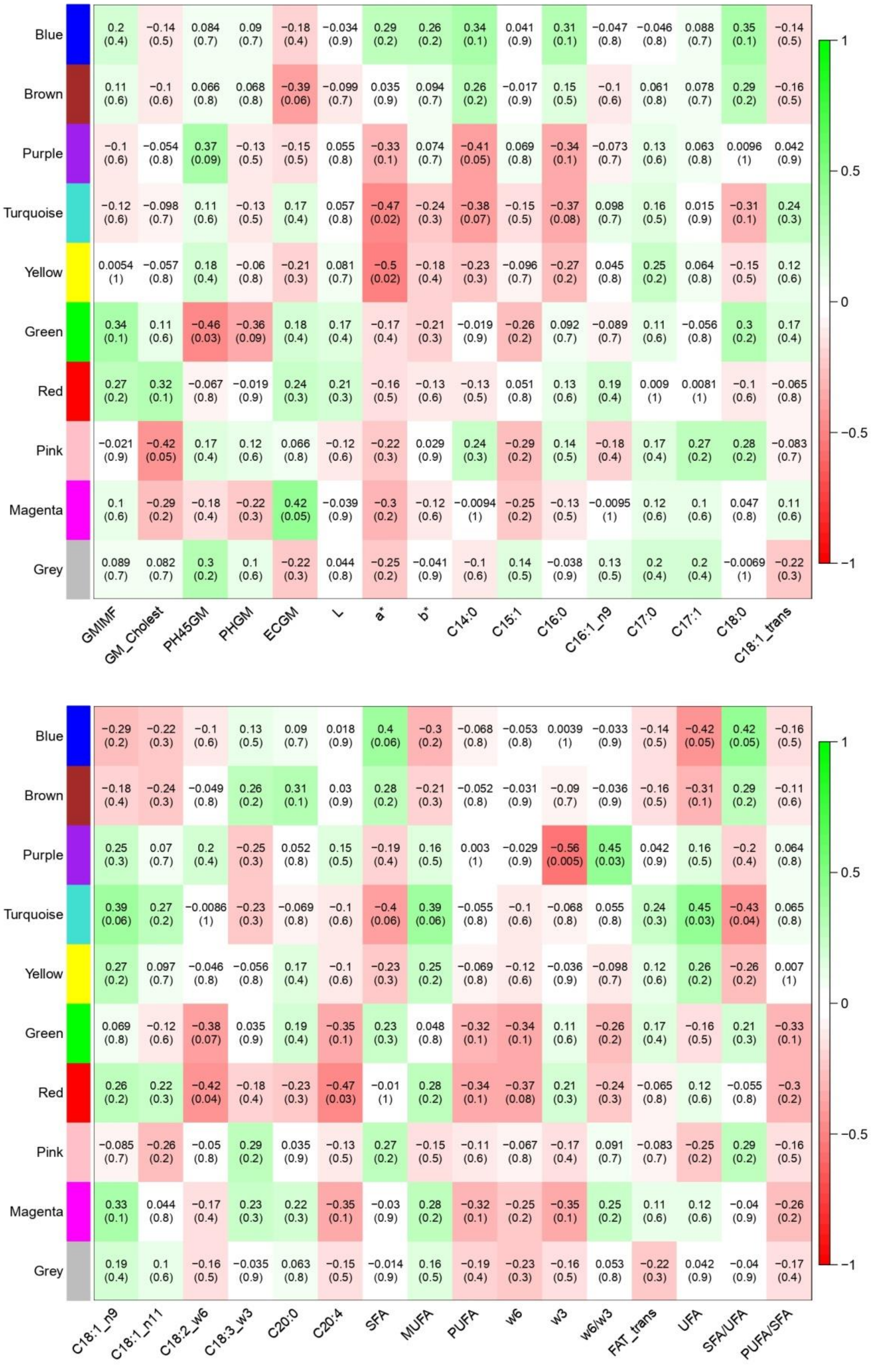

Supplement: Supplementary file 15 — Additional file 15: Figure S1. Sequencing depth obtained for samples analyzed in each one of the two contrasts (AL-T0/AL-T1 and AL-T0/AL-T2). Figure S2. Joint Principal Component Analysis (PCA) clustering of gluteus medius skeletal muscle samples (11 AL-T0, 12 AL-T1 and 12 AL-T2 samples) according to the expression profiles of (A) mRNAs, (B) microRNAs and (C) lincRNAs. Figure S3. Phylogenetically conserved 7mer-8 m predicted binding sites in the 3′- UTR of the pig PDK4 gene for (A) ssc-miR-148a-3p and (B) ssc-miR-493-5p porcine miRNAs. The TargetScan software was used for generating conservation graphs across the investigated mammalian species. Red nucleotides show complementary matching base-pairs between the seed of the mature miRNA and the 3’UTR of the pig PDK4 gene. Figure S4. Gene co-expression module association with meat quality and fatty acids composition traits in the AL-T0/AL-T1 contrast as determined with the WGCNA tool. Figure S5. Gene co-expression module association with meat quality and fatty acids composition traits in the AL-T0/AL-T2 contrast as determined with the WGCNA tool. [file 40104_2019_412_MOESM15_ESM.pdf]
